# Long-term evaluation of safety and biological effects of Korean Red Ginseng (*Panax Ginseng*): a long-term in vivo study

**DOI:** 10.1186/s12906-022-03736-5

**Published:** 2022-11-04

**Authors:** Soo Kyung Park, Sung-Won Kim, Hwi Won Seo, Sun Hee Hyun, Jong-Su Kyung, Soo-Hyun Youn, Seung Ho So, Gyo In, Chae-Kyu Park, Eugene C. Yi, Chang-Kyun Han, Yong Yook Lee

**Affiliations:** 1 Laboratory of Efficacy Research, Korea Ginseng Corporation, 30 Gajeong-ro, Shinseong- dong, Yuseong-gu, 34128 Daejeon, Republic of Korea; 2 Infectious Disease Research Center, Korea Research Institute of Bioscience and Biotechnology (KRIBB), 125 Gwahak-ro, Yuseong- gu, 34141 Daejeon, Republic of Korea; 3 Department of Molecular Medicine and Biopharmaceutical Sciences, Graduate School of Convergence Science and Technology, Seoul National University, 03080 Seoul, Republic of Korea

**Keywords:** Immunity, Korean Red Ginseng, Proteomics, Safety

## Abstract

**Background:**

Although Korean Red Ginseng (KRG) is safe, this finding was only evaluated in 3-mo-long studies. Its safety was verified through a 6-mo KRG administration clinical study, but long-term studies beyond 6 mo are insufficient. This study investigated the safety and efficacy of 12-mo KRG administration.

**Methods:**

In this study, 300 mg/kg of KRG was administered to male and female Sprague Dawley rats for 4, 8, and 12 mo to evaluate its efficacy and safety. Clinical signs, including pathological examination and haematological analyses, were observed. Flow cytometric analyses were utilised to analyse spleen and thymus immune cell counts after 12 mo. Proteomic analysis of the sera was performed using a nanospray-interfaced mass spectrometer with an 11-plex Tandem Mass Tag (TMT) labelling system. Bioinformatic analysis was then performed using Ingenuity Pathway Analysis and PANTHER. Data are available via ProteomeXchange with identifier PXD032036.

**Results:**

No significant body and organ weight changes were observed, and haematological and serum biochemical analyses did not show clinical significance. The effectiveness of long-term KRG administration was confirmed through increased immune cell distribution and activity. Changes in proteins correlated with viral infection reduction were confirmed through proteomic analysis.

**Conclusion:**

The results suggested that 12-mo KRG intake is safe, improves immune system activity, and reduces viral infections with no significant changes in toxicological aspects.

**Supplementary Information:**

The online version contains supplementary material available at 10.1186/s12906-022-03736-5.

## Background

The development of clinical services has led to increased life expectancy in recent decades by controlling diverse diseases [1]. Specifically, the global population’s lifestyle changes and income increased interest in health issues, such as non-communicable diseases. For infectious diseases like COVID-19, the lack of a cure or vaccine motivated consumers to protect themselves and improve their immune systems by adopting functional foods and healthier diets [2]. Notably, the demand for functional foods in the global market, including those with natural products, has increased to help consumers sustain a healthy lifestyle [3]. Food safety has become a significant issue with a growing functional food market because adverse reactions have also increased. Natural product safety is a significant concern because these formulations contain various ingredients, making it difficult to predict their toxicity after administration [4].

Korean Red Ginseng (KRG), or processed *Panax ginseng* Meyer, is indigenous to Korea. It has gained six approvals from the Korean Food and Drug Administration (KFDA) as a functional food for improving immune function, antioxidant activity, memory, blood circulation, and fatigue recovery. Safety studies related to KRG have reported no serious side effects when consuming KRG, noting that symptoms were mild and temporary [5]. However, most KRG safety experiments were only evaluated for short-term safety or 12 wk [5], and only one long-term clinical study evaluated KRG safety for 24 wk [4]. However, studies on more long-term safety are insufficient, and efficacy studies related to this are necessary to ensure the reliability of KRG. Therefore, this study evaluates the long-term safety and efficacy of KRG by administering 300 mg/kg of KRG to Sprague Dawley rats for 4, 8, and 12 mo. The body and organ weight changes were investigated through haematological and serum biochemical analyses and pathological examinations. In addition, proteomic analysis was used to confirm the effectiveness of long-term KRG administration and evaluate immune cell classification and activity. The authors confirm that the study was reported according to the ARRIVE guidelines.

## Materials and methods

### KRG water extract and general chemicals

KRG was prepared using a 6-yr-old ginseng extract prepared by the Korea Ginseng Corporation following the ISO 19610 international standard production process. Ammonium bicarbonate (ABC), dithiothreitol (DTT), iodoacetamide (IAA), and urea were purchased from Merck (MO, USA). Meanwhile, trypsin (MS grade) was obtained from Thermo Fisher Scientific (IL, USA).

### Long-term safety study

#### Test conditions and experimental specimens

**Fig. 1:**
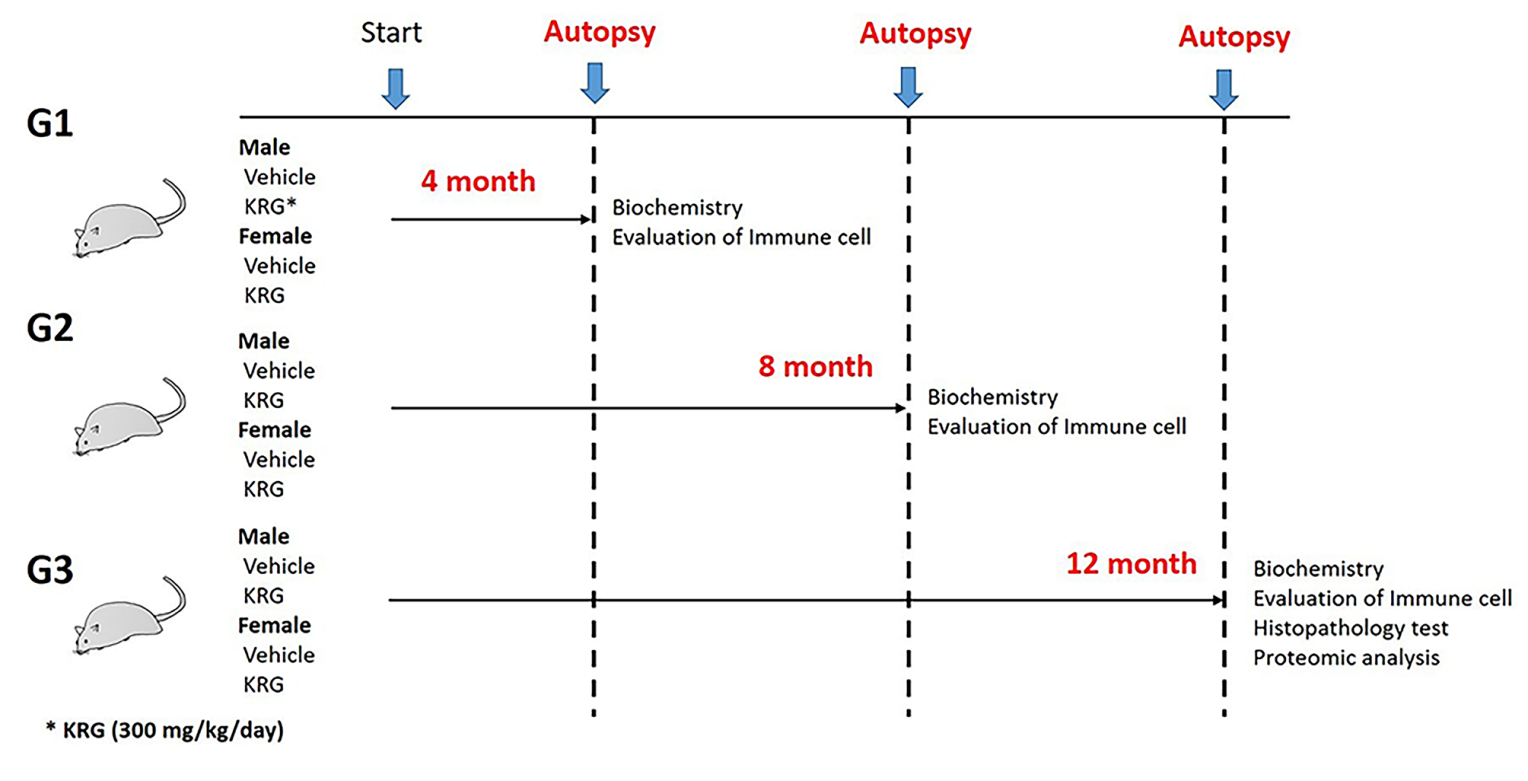
Schematic chart of Korean Red Ginseng (KRG) administration. Rats were divided into 3 groups (10 males or females per group) per duration (G1: 4 mo, G2: 8 mo, G3: 12 mo). The KRG groups received 300mg/kg doses per day

A total of 120 4-wk-old male and female, specific pathogen-free (SPF) Sprague-Dawley (SD) rats were obtained from Koatech Inc. (Pyeongtaek-si, Korea). Before administration, the rats were acclimatised for 7 d by being housed in polycarbonate plastic cages (1 rat per cage) with aspen porous good laboratory practice (GLP) bedding (Samtako, Osan-si, Korea) in a room with controlled temperature (22°C ± 3°C), humidity (50%, 50% ± 20%), and a 12-h light/dark cycle. The rats were fed with standard rodent chow (Purina, USA) and filtered water ad libitum. At five weeks old, the rats were divided into 12 groups randomly (10 males and 10 females per group), which included male and female vehicle controls (distilled water) and a KRG-treated group (300 mg/kg/day). Figure 1 shows the groups used in the study, which mainly consist of 4 groups (2 control and 2 KRG groups for males and females) for each duration (4, 8, and 12 mo).

#### Body and organ weight changes

The rats’ body weights were measured weekly from the first day of administration to the day of necropsy. Organs, including the brain, heart, lung, liver, spleen, kidney, adrenal gland, testis, and uterus, were dissected, and their absolute weights were measured. Relative organ weight was calculated as the ratio between the absolute organ weight and the body’s weight before necropsy.

#### Food consumption changes

Food consumption was measured weekly during the mid and late experimental study from weeks 18 to 50. Daily food consumption (g/rat/day) was calculated as the difference between the newly supplied food and the remaining food for the next day.

### Analytical tests

#### Biochemistry and haematology

Before necropsy, all rats were fasted overnight and euthanised by exsanguination under isoflurane anaesthesia after recording their body weights. Blood samples were drawn from the inferior vena cava, collected in ethylenediaminetetraacetic acid (EDTA) vacutainers, and analysed for white blood cell (WBC) count, red blood cell (RBC) count, haemoglobin (Hb), haematocrit (Hct), mean corpuscular volume (MCV), mean corpuscular haemoglobin (MCH), mean corpuscular haemoglobin concentration (MCHC), and platelet (PLT) count using a blood cell counter (MS9-5, Melet Schloesing Laboratories, France). For clinical biochemistry analyses, serum was obtained from blood collected in EDTA-free vacutainers by centrifugation at 3,000rpm for 10min. An automatic biochemical analyser (7100, HITACHI, Japan) was used to analyse the following in the blood samples: alanine aminotransferase (ALT), aspartate aminotransferase (AST), alkaline phosphatase (ALP), blood urea nitrogen (BUN), creatinine (CREA), total protein (TP), albumin (ALB), albumin-globulin (A/G) ratio, total cholesterol (T-CHO), triglyceride (TG), and glucose (GLU). The remnant serum was stored at − 78°C until the global proteomic profiling analysis was performed.

#### Flow cytometry analysis for immune cells 12-months KRG administration

After the rats were euthanised after 12 mo of administration, their spleens and thymi were collected and placed in an ice-cooled Roswell Park Memorial Institute (RPMI) medium. The prepared spleens were filtered with a 40 mm nylon mesh, the supernatant was removed by centrifugation, and 2 mL of ACK lysis buffer was added. Then, 5 mL of RPMI 1640 medium was added and centrifugally washed twice (1200 rpm, 5 min, 4°C). The procedure for flow cytometric analysis follows the method of Kim et al. [6]. During analysis of the population of immune cells, the cells were washed with FBS strain buffer (BD Pharmingen™) and incubated with anti-rat CD32 for 20min at 4°C to prevent nonspecific binding.

The cells were incubated with the following fluorochrome-conjugated antibodies specific for FITC Mouse Anti-Rat CD45R, PE Mouse Anti-Rat CD4, PerCP Mouse Anti-Rat CD8a, FITC Mouse Anti-Rat Granulocytes, PE Mouse Anti-Rat RT1B, PE-Cy™5 Mouse Anti-Rat CD4, FITC Mouse Anti-Rat CD25, FITC Mouse Anti-Rat CD161a, PE Mouse Anti-Rat IFN-γ (BD Bioscience), CD3 Monoclonal Antibody (Invitrogen), and PE anti-mouse/rat/human FOXP3 Antibody (Biolegend). Next, the samples were washed with phosphate-buffered saline (PBS) 3 times. CytoFLEX (Beckman Coulter, Brea, CA, USA) was used to analyse the resulting samples using the stained cells per 10,000 cells. The data were analysed using FlowJo software (Tree Star Software, San Carlos, California, USA).

#### Histopathology

Each rat’s dissected organs were trimmed and observed macroscopically after 12 mo of KRG administration. The heart, lung, liver, spleen, and kidneys were fixed in 10% neutral buffered formalin. After paraffin infiltration using a tissue processor, the organs were embedded in paraffin, cut into 4 μm sections, stained with haematoxylin and eosin, and examined under a light microscope (Olympus BX50, Japan).

### Statistical analysis

The results are presented as the mean ± SEM. Data from body weights, food consumption, organ weights, blood biochemistry, and haematology indices were analysed using a student’s t-test or the Mann-Whitney test of the SPSS software program. The results were considered statistically significant at a value of *p* < 0.05.

### Global proteomic profiling analysis

#### Immunodepletion of abundant proteins with immunoglobulin Y (IgY)-R7 cartridge

Six biological replicates among the 12 mo KRG administration and control groups were individually prepared for all six conditions, following the manufacturer’s instructions. For each serum sample, immunodepletion of the seven most abundant proteins (serum albumin, immunoglobulin G (IgG), fibrinogen, transferrin, immunoglobulin M (IgM), haptoglobin, and alpha1-antitrypsin) in rat serum was performed with a Seppro IgY-R7 spin cartridge (SEP130, Merck), containing most of the immobilised antibodies against the seven proteins. The collected flow-through sample’s seven-protein concentration was determined using a bicinchoninic acid (BCA) assay (Thermo Fisher Scientific, IL, USA).

#### Protein digestion and TMT 11-plex labelling

Each depleted serum sample was denaturised with 8M urea in 50 mM ammonium bicarbonate (ABC). Then, dithiothreitol (DTT) was added to each sample (now at 10 mM) and incubated for 1h at 37°C, and IAA was added (now at 40 mM) and incubated at the same temperature and duration. The sample was diluted with 50 mM ABC (pH 8.5) to become 1 M urea, and 1 mM calcium chloride (CaCl_2_) and trypsin at a 1:50 trypsin to sample ratio (w/w) were added and incubated overnight at 37°C. The peptide sample was desalted using a C18 SPE cartridge (Merck) and subjected to a BCA assay. A subfraction (about 3 µg of peptide) from each sample was mixed into a single tube to prepare the pooled reference sample.

Four sets of samples using Tandem Mass Tag (TMT, Thermo Fisher Scientific) 11-plex labelling were prepared for system-wide quantitative comparison. Briefly, 5 µL of the peptide in each sample was obtained and labelled with the adequate TMT 11-plex reagent channel according to the manufacturer’s protocols. The TMT-labelled peptide samples in the same set were then combined (for a total of 55 µL of peptide sample) for further fractionation.

#### Multidimensional mid-ph (pH 8) reverse-phase liquid chromatography (RPLC) fractionation

In this step, microscale (25 µg) mid-pH reverse-phase liquid chromatography RPLC fractionation was performed using an in-house packed 200 μm I.D. A C18 capillary column nanoACQUITY ultra-performance liquid chromatography (UPLC; Waters) was utilised with a 3 µL/min flow rate. Meanwhile, using a linear gradient of solvent A (10 mM ABC in water) and solvent B (10 mM ABC in 90% acetonitrile (I)) caused an increase from 2 to 40% in the latter for 90min. The eluent was automatically collected and concatenated into 12 fractions using Triversa NanoMate (Advion, NY, USA).

#### LC-MS3 experiment

All four TMT-labeled plasma sample sets were analysed using an Orbitrap Fusion Lumos Tribrid mass spectrometer (MS; Thermo Fisher Scientific) in data-dependent synchronous precursor selection (SPS)-MS3 mode. The mass spectrometer was coupled with nanoACQUITY UPLC equipped with an in-house packed trap (150μm I.D. x 3cm) and analytical column (75 μm I.D. x 100 cm) using 3μm Jupiter C18 column particles (Phenomenex). In this step, the 10 most intense ions were isolated at 0.5 Thomson (Th) of precursor isolation width under an identical full MS scan setting for collision-induced dissociation (CID) with tandem mass spectrometry (MS/MS) in an ion trap with 4E3 of automatic gain control (AGC), 150 ms of maximum injection time (ITMax), and wideband activation. The synchronous precursor selection (SPS)-based higher-energy collision dissociation (HCD) MS3 was performed by isolating the 10 most intense MS2 fragment ions using 2 m/z of isolation width, 1.5E5 of AGC, 250 ms of ITMax, and 55% of normalised collision energy (NCE).

#### Database search, quantitative analysis, and bioinformatic analysis

For the quantitative analysis of TMT-labeled rat plasma datasets, Proteome Discoverer (PD) ver. 2.3 (Thermo Fisher Scientific) was cross-referenced against the UniProt rat reference proteome database (29,944 entries) using the following parameters: static carbamidomethylation of cysteine, dynamic oxidation of methionine, and dynamic TMT11 modification of peptide N-terminus and lysine. The resulting peptide hits were filtered with a 1% maximum false discovery rate (FDR) using the Percolator algorithm. The reporter ion ratios reported from PD were adjusted by applying the isotopic correction factors of the manufacturer’s TMT kit. Only reporter ions containing spectra were designated as “quantifiable spectra.” CID-MS2 spectra were used for peptide identification in this step, whereas HCD-MS3 spectra containing reporter ion signals were used for quantification. Based on the data, the evaluation of biological functions was performed using Ingenuity Pathway Analysis (IPA; Ingenuity Systems; Redwood City, CA, USA) and the PANTHER classification system.

## Results

### Body and organ weight changes

A total of 300 mg/kg of KRG was administered to Sprague Dawley rats for up to 12 mo. During the exposure period, noticeable mortality and clinical signs were not observed, and there were no statistically significant body weight changes (Fig. S1). Aside from a decrease in the ovary’s weight, significant organ weight changes were not observed in relative organ weights (Table S1).

### Food consumption changes

During the food consumption monitoring period from weeks 18 to 50, there were no significant differences in food consumption between KRG treatment and control groups for both male and female specimens (Fig. S2).

### Haematology and biochemistry

**Fig. 2:**
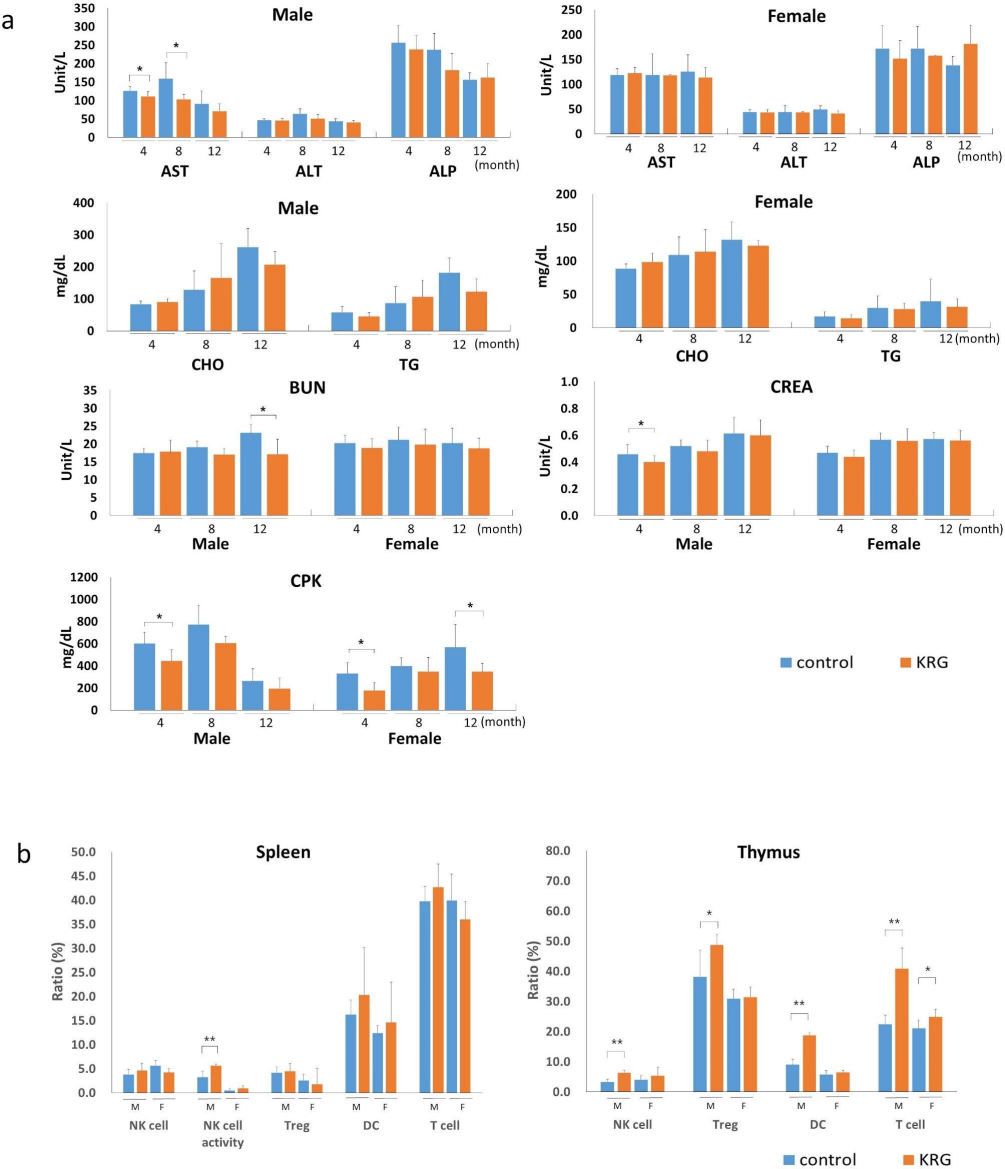
Blood biochemistry data and immune cell count changes for rats orally administered with Korean Red Ginseng (KRG). (**a**) Biochemical parameters of liver functions (aspartate transaminase (AST, U/L), alanine aminotransferase (ALT, U/L), and Alkaline phosphatase (ALP, U/L)); lipid parameters (total cholesterol (CHO, mg/dL), triglyceride (TG, mg/dL); the parameters of kidney functions (blood urea nitrogen (BUN, mg/dL), creatinine (CREA, mg/dL); and creatine phosphokinase (CPK, mg/dL) in KRG administered male and female rats. Results are expressed as mean ± SD (n = 8). (**b**) Changes in immune cell count for 12 mo KRG administration, showing splenic and thymic immune cell counts. Significant difference vs. control *(*p* < 0.05), **(*p* < 0.01)

There were no significant differences between the control and KRG-treated groups in the haematology analyses. MCV and MCHC significantly increased (*p* < 0.05) in the 8 mo administration group’s female rats than in the control group. However, WBC count significantly decreased (*p* < 0.05) in the male rats of the KRG group compared to the control group (Table 1). The AST levels in the male KRG administration group at 4 and 8 mo decreased significantly compared to the control group (*p* < 0.05), and there was no significant difference in the female KRG administration group. Meanwhile, there was no significant difference in both male and female KRG administration groups at 12 mo. (Fig. 2a) CHO and TG levels tended to decrease compared to the control group, but there was no significant difference. However, BUN in the 12 mo and CREA in the 4 mo male KRG administration group decreased significantly (*p* < 0.05) compared to the control group. In addition, the CPK level was significantly lower (*p* < 0.05) in the 4 mo old male and female groups and the 12 mo old female KRG administration group (*p* < 0.05).

**Table 1:** Haematological data for 4, 8, and 12 mo KRG administration (n = 8, mean ± SD)

Month	Dose (mg/kg)	WBC(K/μL)	LYM(K/μL)	MON(K/μL)	NEU(K/μL)	EO(K/μL)	BA(K/μL)	RBC(M/μL)	MCV(fL)	HCT(%)	MCH(pg)	MCHC(g/dL)	Hb(g/dL)	RDW-SD(fL)	PLT(K/μL)	MPV(fL)
Male																
4	Control	5.27 ± 1.07	3.33 ± 0.57	0.54 ± 0.23	0.93 ± 0.47	0.13 ± 0.05	0.20 ± 0.05	8.03 ± 0.44	49.57 ± 1.22	39.80 ± 1.84	18.44 ± 0.52	37.20 ± 1.05	14.78 ± 0.59	33.56 ± 0.50	649 ± 163	6.15 ± 0.54
	300	5.50 ± 1.31	3.57 ± 0.73	0.57 ± 0.29	0.89 ± 0.41	0.11 ± 0.06	0.19 ± 0.06	7.91 ± 0.37	49.27 ± 2.07	38.91 ± 1.13	18.36 ± 0.78	37.25 ± 0.51	14.50 ± 0.40	33.49 ± 0.71	743 ± 58	6.09 ± 0.47
8	Control	4.09 ± 1.04	2.03 ± 0.34	0.30 ± 0.11	1.15 ± 0.73	0.00 ± 0.00	0.20 ± 0.11	8.27 ± 0.25	51.65 ± 1.66	42.70 ± 1.95	17.63 ± 0.59	34.13 ± 0.64	14.58 ± 0.46	34.60 ± 0.39	780 ± 47	6.17 ± 0.14
	300	3.67 ± 0.72	1.73 ± 0.33	0.27 ± 0.08	1.05 ± 0.42	0.07 ± 0.05	0.20 ± 0.06	8.22 ± 0.23	51.18 ± 1.50	42.02 ± 0.91	17.63 ± 0.56	34.43 ± 0.56	14.48 ± 0.28	34.43 ± 0.29	804 ± 140	6.13 ± 0.22
12	Control	6.52 ± 1.66	1.82 ± 0.89	3.27 ± 0.76	0.47 ± 0.14	0.07 ± 0.08	0.30 ± 0.06	8.16 ± 0.41	13.78 ± 0.78	44.98 ± 3.50	55.10 ± 2.40	16.93 ± 0.40	30.75 ± 0.98	17.60 ± 0.52	860 ± 83	6.20 ± 0.31
	300	4.12 ± 1.02*	1.23 ± 0.38	2.10 ± 0.51	0.37 ± 0.12	0.00 ± 0.00	0.18 ± 0.04	8.26 ± 0.21	13.98 ± 0.66	45.23 ± 2.18	54.77 ± 2.18	16.95 ± 0.65	30.97 ± 0.28	17.85 ± 0.29	759 ± 150	6.47 ± 0.34
Female																
4	Control	4.23 ± 1.00	3.09 ± 0.78	0.24 ± 0.11	0.37 ± 0.17	0.19 ± 0.11	0.13 ± 0.05	6.78 ± 1.21	55.54 ± 6.29	36.98 ± 3.93	22.08 ± 5.26	39.32 ± 4.46	14.40 ± 0.59	34.45 ± 2.49	575 ± 88	5.93 ± 0.25
	300	5.08 ± 1.49	3.70 ± 1.14	0.32 ± 0.10	0.52 ± 0.20	0.22 ± 0.20	0.15 ± 0.07	7.16 ± 0.53	54.16 ± 2.09	38.74 ± 2.52	20.37 ± 1.81	37.57 ± 2.23	14.50 ± 0.60	33.66 ± 0.95	619 ± 111	5.79 ± 0.32
8	Control	3.52 ± 1.59	2.17 ± 0.99	0.18 ± 0.10	0.68 ± 0.46	0.13 ± 0.15	0.17 ± 0.08	8.02 ± 0.33	59.77 ± 1.26	47.90 ± 2.22	16.75 ± 0.63	27.98 ± 0.65	13.40 ± 0.53	35.32 ± 0.88	838 ± 158	5.93 ± 0.35
	300	2.81 ± 1.03	1.72 ± 0.50	0.15 ± 0.05	0.57 ± 0.41	0.10 ± 0.11	0.13 ± 0.05	8.09 ± 0.51	55.82 ± 3.66*	45.10 ± 2.51	16.53 ± 0.88	29.70 ± 1.76*	13.37 ± 0.67	35.47 ± 1.17	818 ± 146	6.15 ± 0.24
12	Control	2.64 ± 0.64	0.74 ± 0.36	1.38 ± 0.32	0.21 ± 0.08	0.00 ± 0.00	0.11 ± 0.04	7.54 ± 0.48	13.08 ± 0.61	42.60 ± 1.94	56.56 ± 2.62	17.38 ± 0.81	30.71 ± 0.64	16.98 ± 0.78	712 ± 168	6.16 ± 0.39
	300	2.49 ± 0.42	0.68 ± 0.16	1.36 ± 0.23	0.19 ± 0.04	0.00 ± 0.00	0.10 ± 0.00	7.31 ± 1.32	12.61 ± 1.95	41.34 ± 6.01	57.10 ± 3.95	17.36 ± 0.95	30.45 ± 0.59	17.18 ± 0.33	760 ± 77	6.08 ± 0.35

### Flow cytometry analysis for immune cells

Between the 12 mo KRG administration group and the control group, immune cell counts in rats were investigated to evaluate the immunological effects of long-term KRG administration (Fig. 2b). Flow cytometric analysis showed that after 12 mo of KRG administration, the ratio of thymic immune cells, including NK cells, dendritic cells (*p* < 0.01) and Treg cells (*p* < 0.05) in male rats, and T cells in both males (*p* < 0.01) and females (*p* < 0.05) significantly increased (Fig. 2b). Meanwhile, the splenic cell ratio increased, but there was no significant statistical significance. However, NK cell activity in the male spleen significantly increased (*p* < 0.01).

### Histopathology

**Fig. 3:**
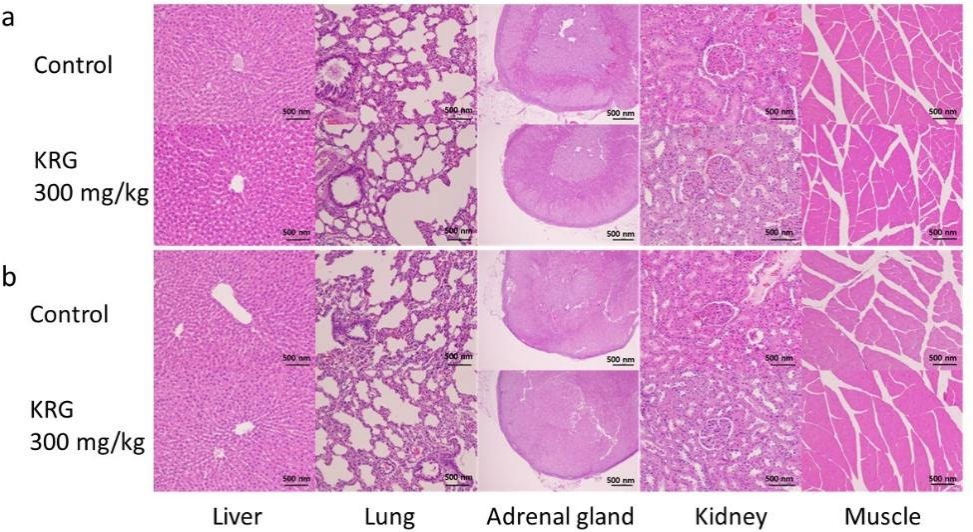
Histopathological changes in rats exposed to 12 mo KRG. (**a**) Male rats. (**b**) Female rats. (Haematoxylin and eosin, magnification: 200X, n = 3)

A histopathological examination was performed between the 12 mo KRG administration and control groups using haematoxylin and eosin (H & E)-stained samples (n = 3). Scores were assigned according to the diagnosed degree’s grade, and numerical comparisons were performed with the average value (grade: minimal, slight, moderate, severe) to facilitate group-by-group comparison. Compared to the control group, all toxicity-related results in the KRG administration tests for the male and female administration groups were identical (Fig. 3). Although spontaneous or ageing-related findings were observed in the male and female administration groups, similar severity and incidence results were observed in the control group. Because the findings were localised or unilateral, both were independent of KRG administration (Table S2).

### Total proteomic analysis for identification of function

**Fig. 4:**
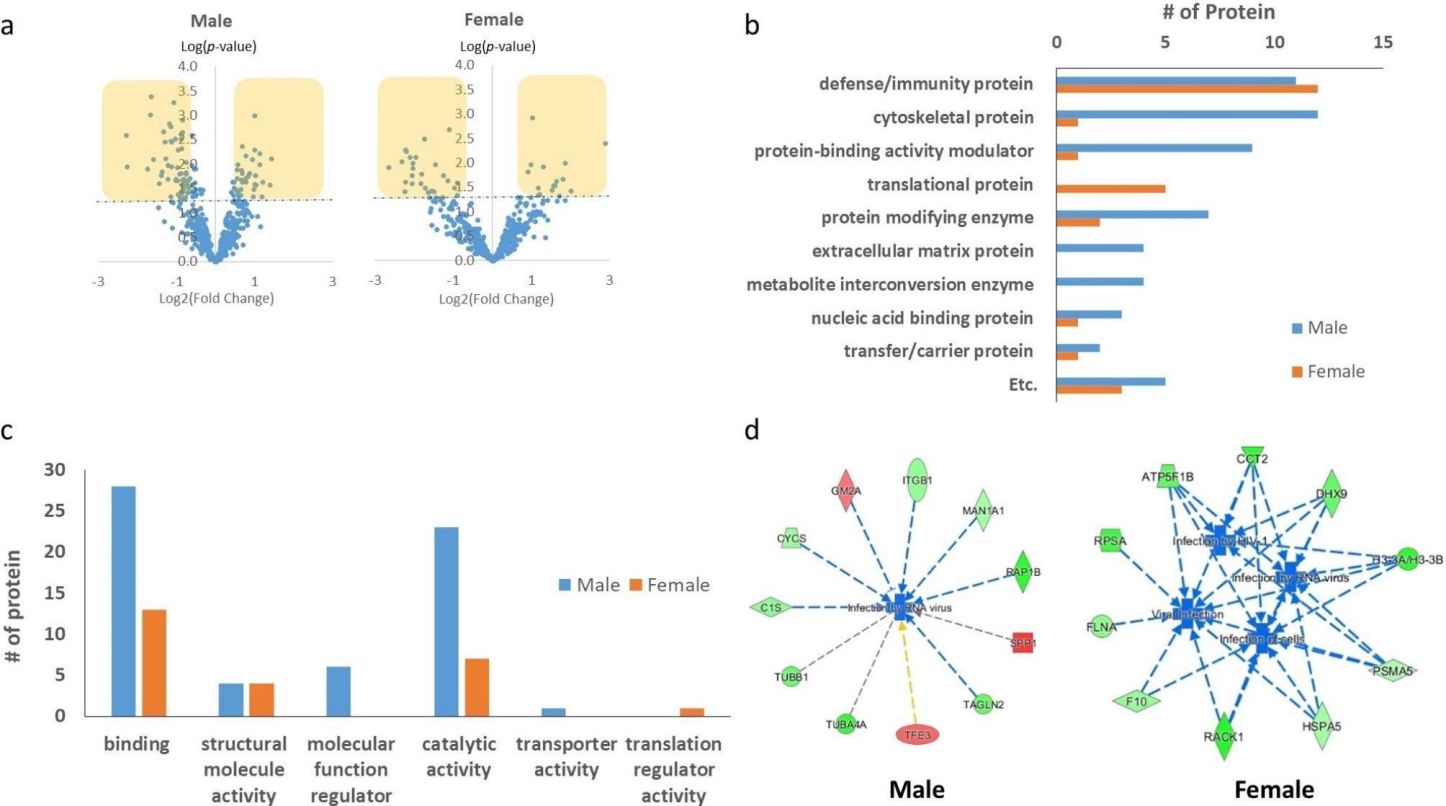
Differentially expressed proteins (DEPs) in rate exposed to Korean Red Ginseng (KRG) and bioinformatic analysis after 12 mo KRG administration (n = 6). (**a**) Volcano plot showing the *p*-value (-log_10_) versus fold change (log_2_) of the proteomic analysis; (**b**) protein class and (**c**) molecular function analysis of DEPs by the Panther classification system; (**d**) selected decreased function of the DEPs in rats administered with KRG for 12 mo by IPA functional analysis (Red: biological process or disease in an upward trend; Green: biological process or disease is trending downward)

Global proteomic profiling analysis of rat sera was performed to identify differentially expressed proteins (DEPs) in rats administered with KRG for 12 mo (n = 6). For quantitative analyses with protein identification, sera samples were labelled using 11-plex TMT agents, and the total identified protein list was compared with a 1% false discovery rate (FDR) level. A total of 493 unique proteins were identified through MS analysis for the male group and 466 for the female group using the target-decoy database (UniProt rat database) of Proteome Discoverer (Fig. 4a).

Next, the differentially expressed proteins (DEPs) with criteria under a level of *p <* 0.05 (T-test) were selected (Table 2). Among the representative proteins chosen in the male rats, GM2A and SPP1 were upregulated, and SERPINA6, and TAGLN2 were downregulated. Otherwise, immunoglobulin (Ig)-like domain-containing proteins and Tf (HGNC:11,740) increased, and F10 (HGNC:3528) and FLNA (HGNC:3754) were lower in the female group. To identify the class and molecular functions of DEPs, a classification analysis of the proteins was performed using the PANTHER tool, which revealed that the proteins are classified into defence/immunity proteins (11), cytoskeletal proteins (12), and protein-biding activity modulators (9) in the male sera. In the female group, the proteins are defence/immunity proteins (12), while the translational proteins were the most highly ranked (Fig. 4b). In addition, most proteins were associated with binding, catalytic, or structural molecule activities shown in (Fig. 4c). To determine disease and function, the pool of DEPs (*p* < 0.05, log_2_ value (|log_2_x| > 0.7) were selected and analysed using IPA. In this step, the male rats’ selected DEPs showed the upregulation of neutrophil movement and downregulation of viral infections. Simultaneously, the female DEPs showed the downregulation of viral infections and tumour cell line viability (Fig. 4d). These results implied that long-term KRG administration protects the male and female rats from viral infections.

**Table 2:** Differentially expressed up or downregulated proteins from male (**a**) and female (**b**) rat sera

**(a)**						
Description	UniProt ID	Gene	Location	Type(s)	Log_2_ FC	***p*** - value
H2A clustered histone 6	Q4FZT6	H2AC6	Nucleus	other	1.436	0.008
Heterogeneous nuclear ribonucleoprotein C, isoform CRAa	A0A0G2JXW4				1.388	0.026
Ig-like domain-containing protein	D3ZEP5				1.201	0.023
Ig-like domain-containing protein	D3ZAB3				1.178	0.046
Secreted phosphoprotein 1	P08721	SPP1	ES	cytokine	1.139	0.006
Tsukushi, small leucine-rich proteoglycan	Q6QMY6	TSKU	ES	other	1.126	0.010
Ig kappa chain V-IV region-like	F1M1R0	LOC690813	Other	other	1.037	0.026
Urinary protein 2	P81828				1.027	0.017
Urinary protein 3-like	M0R7P3				1.002	0.001
Ig-like domain-containing protein	A0A0G2JXB7				1.001	0.005
Urinary protein 2	P81827	LOC100360095 (includes others)	Other	other	0.994	0.026
H1.2 linker histone, cluster member	A0A0G2K654	H1f2	Nucleus	other	0.984	0.032
Ig-like domain-containing protein	M0R7Q2				0.96	0.047
Cochlin	B1H259	Coch	Cytoplasm	other	0.94	0.008
Transcription factor binding to IGHM enhancer 3	D3ZAW6	TFE3	Nucleus	TR	0.869	0.015
Insulin-like growth factor-binding protein 6	P35572	IGFBP6	ES	other	0.835	0.018
Angiogenin	Q5GAM5	ANG	ES	enzyme	0.776	0.007
GM2 ganglioside activator	Q6IN37	GM2A	Cytoplasm	enzyme	0.772	0.023
CD5 molecule like	Q4KM75	CD5L	PM	Tm. Rc.	0.768	0.020
Similar to BC049975 protein	F1M8F5				-0.71	0.039
Mannosidase alpha class 1A member 1	A0A0G2JW29	MAN1A1	Cytoplasm	enzyme	-0.732	0.026
Uncharacterised protein	M0R5J4				-0.746	0.015
Filamin A	C0JPT7	FLNA	Cytoplasm	other	-0.754	0.016
NME/NM23 nucleoside diphosphate kinase 2	P19804	NME2	Nucleus	kinase	-0.76	0.026
Ig-like domain-containing protein	M0RDF2				-0.762	0.009
Paraoxonase 1	P55159	PON1	ES	phosphatase	-0.764	0.048
Serpin family D member 1	A0A0G2K8K3	SERPIND1	ES	other	-0.786	0.026
RIKEN cDNA 1300017J02 gene	E9PST1	1300017J02Rik	ES	other	-0.811	0.022
Cytochrome c, somatic	P62898	CYCS	Cytoplasm	transporter	-0.812	0.020
Tyrosine 3-monooxygenase/tryptophan 5-monooxygenase activation protein zeta	A0A0G2JV65	YWHAZ	Cytoplasm	enzyme	-0.818	0.027
Tropomyosin 3	Q63610	Tpm3	Cytoplasm	other	-0.821	0.034
Histidine-rich glycoprotein	A0A0G2K3G0	Hrg	Cytoplasm	other	-0.824	0.050
Serpin family A member 6	P31211	SERPINA6	ES	other	-0.837	0.033
Profilin 1	P62963	PFN1	Cytoplasm	other	-0.845	0.021
Integrin subunit beta 1	A0A0G2JSK5	ITGB1	PM	Tm. Rc.	-0.85	0.001
Serine protease 3	D3ZQV0	PRSS3	ES	peptidase	-0.852	0.002
Caveolae associated protein 2	Q66H98	CAVIN2	PM	other	-0.862	0.040
Cofilin 1	P45592	CFL1	Nucleus	other	-0.863	0.005
Carboxylesterase 1C	D3ZGK7	Ces1c	ES	enzyme	-0.866	0.027
Complement component 4 binding protein alpha	Q63514	C4BPA	ES	other	-0.878	0.003
Phosphoglycerate kinase 1	P16617	PGK1	Cytoplasm	kinase	-0.88	0.002
FYN binding protein 1	D3ZIE4	FYB1	Nucleus	other	-0.883	0.037
Tubulin beta class I	P69897	TUBB	Cytoplasm	other	-0.888	0.015
Thymosin beta 4 X-linked	P62329	TMSB10/TMSB4X	Cytoplasm	other	-0.907	0.047
Complement C1s	G3V7L3	C1S	ES	peptidase	-0.911	0.049
Alpha-1-B glycoprotein	Q9EPH1	A1BG	ES	other	-0.921	0.016
Carboxylesterase 1C	P10959	Ces1c	ES	enzyme	-0.951	0.043
Inter-alpha-trypsin inhibitor heavy chain 4	Q5EBC0	ITIH4	ES	other	-0.964	0.005
Serglycin	P04917	Srgn	Cytoplasm	other	-0.981	0.012
Cysteine- and glycine-rich protein 1	P47875	CSRP1	Nucleus	other	-0.984	0.022
Haemoglobin subunit beta-2-like	Q62669	LOC103694855	Other	other	-1.014	0.020
Glutathione peroxidase 3	A0A0G2K531	GPX3	ES	enzyme	-1.02	0.022
Thioredoxin	R4GNK3	TXN	Cytoplasm	enzyme	-1.034	0.003
Ig-like domain-containing protein	F1LWD0				-1.072	0.001
Pleckstrin	A0A0G2K393	PLEK	Cytoplasm	other	-1.119	0.003
Cyclase associated actin cytoskeleton regulatory protein 1	Q08163	CAP1	PM	other	-1.131	0.011
Phospholipid transfer protein	E9PSP1	PLTP	ES	enzyme	-1.151	0.047
Ceruloplasmin	G3V7K3	CP	ES	enzyme	-1.152	0.002
Tropomyosin 4	P09495	Tpm4	Cytoplasm	other	-1.153	0.003
Serpin family A member 10	Q62975	SERPINA10	ES	other	-1.178	0.001
Actin beta	A0A0G2K3K2	ACTB	Cytoplasm	other	-1.211	0.020
Tubulin beta 1 class VI	M0R8B6	TUBB1	Cytoplasm	other	-1.217	0.011
Transgelin 2	Q5XFX0	TAGLN2	Cytoplasm	other	-1.269	0.008
Carboxypeptidase N subunit 2	F1LQT4	CPN2	ES	peptidase	-1.297	0.004
Complement component 4 binding protein beta	A0A5C5	C4BPB	ES	other	-1.334	0.002
Ig-like domain-containing protein	F1LYQ4				-1.365	0.014
Calponin 2	D3ZRX9	CNN2	Cytoplasm	other	-1.389	0.017
Deleted in malignant brain tumours 1	Q8CIZ5	DMBT1	PM	Tm. Rc.	-1.435	0.025
Lactate dehydrogenase A	P04642	Ldha/RGD1562690	Cytoplasm	enzyme	-1.597	0.008
Tubulin alpha 4a	Q5XIF6	TUBA4A	Cytoplasm	other	-1.655	0.000
RAP1B, member of RAS oncogene family	Q62636	RAP1B	Cytoplasm	enzyme	-1.671	0.001
Complement C8 beta chain	P55314	C8B	ES	other	-1.743	0.013
Actin alpha 1, skeletal muscle	P68136	ACTA1	Cytoplasm	other	-2.257	0.011
H2A.Z variant histone 2	D4AEC0	H2AZ2	Nucleus	other	-2.287	0.003
**(b)**						
**Description**	**UniProt ID**	**Gene**	**Location**	**Type(s)**	**Log10 FC**	***p*** **-value**
Ig-like domain-containing protein	M0RD98				2.871	0.004
Ig-like domain-containing protein	M0RDL2				2.002	0.037
Ig-like domain-containing protein	M0R8G6				1.849	0.010
Ig-like domain-containing protein	F1LTY5				1.791	0.022
Ig-like domain-containing protein	F1M2W3				1.708	0.046
Immunoglobulin kappa constant	P01836	Igkc	ES	other	1.621	0.024
Ig-like domain-containing protein	D3ZWC1				1.591	0.029
Ig-like domain-containing protein	F1LW26				1.549	0.028
Ig-like domain-containing protein	D3ZEP5				1.521	0.036
Keratin 86	A0A0G2QC11	KRT86	Cytoplasm	other	1.301	0.012
Ig-like domain-containing protein	A0A0G2K7S9				1.194	0.044
Ig-like domain-containing protein	M0RBK4				1.16	0.032
Keratin 87	A7M746	Krt87	ES	other	1.154	0.045
Lipopolysaccharide binding protein	Q63313	LBP	PM	transporter	1.02	0.001
Insulin-like growth factor-binding protein 4	P21744	IGFBP4	ES	other	0.984	0.050
Glycoprotein V platelet	G3V9H9	NEWGENE_2724	Other	other	0.976	0.010
Transferrin	P12346	TF	ES	transporter	0.904	0.015
Carboxypeptidase B2	Q9EQV9	CPB2	ES	peptidase	0.719	0.049
Vinculin	A0A0G2K8V2	VCL	PM	enzyme	-0.901	0.045
Proteasome 20S subunit alpha 5	P34064	PSMA5	Cytoplasm	peptidase	-0.906	0.025
Heat shock protein family A (Hsp70) member 5	P06761	HSPA5	Cytoplasm	enzyme	-0.989	0.032
Proteasome 20S subunit beta 1	P18421	PSMB1	Cytoplasm	peptidase	-1.11	0.002
Coagulation factor X	Q63207	F10	ES	peptidase	-1.147	0.023
Filamin A	C0JPT7	FLNA	Cytoplasm	other	-1.266	0.018
Peroxiredoxin 5	Q9R063	PRDX5	Cytoplasm	enzyme	-1.449	0.050
Thymosin beta 4 X-linked	P62329	TMSB10/TMSB4X	Cytoplasm	other	-1.472	0.011
Ig-like domain-containing protein	F1LWW1				-1.515	0.039
Complement C8 beta chain	P55314	C8B	ES	other	-1.561	0.037
DExH-box helicase 9	D4A9D6	DHX9	Nucleus	enzyme	-1.655	0.040
ATP synthase F1 subunit beta	P10719	ATP5F1B	Cytoplasm	transporter	-1.731	0.032
Lactate dehydrogenase A	P04642	Ldha/RGD1562690	Cytoplasm	enzyme	-1.756	0.003
40S ribosomal protein S26	A0A0G2JY64				-1.831	0.017
Uncharacterised protein	D3ZM33				-1.839	0.026
Tropomyosin 4	P09495	Tpm4	Cytoplasm	other	-1.971	0.007
Ribosomal protein L15	P61314	RPL15	Cytoplasm	other	-2.041	0.013
Prohibitin	P67779	PHB	Nucleus	TR	-2.05	0.010
Ribosomal protein SA	P38983	RPSA	Cytoplasm	TR	-2.062	0.018
Ribosomal protein L18a	P62718	RPL18A	Cytoplasm	other	-2.065	0.026
X-ray repair cross-complementing 6	Q6AZ64	XRCC6	Nucleus	enzyme	-2.12	0.007
Eukaryotic translation initiation factor 4A1	Q6P3V8	EIF4A1	Cytoplasm	TR	-2.213	0.006
Chaperonin containing TCP1 subunit 2	Q5XIM9	CCT2	Cytoplasm	kinase	-2.231	0.025
Ubiquitin A-52 residue ribosomal protein fusion product 1	P62986	Uba52	Cytoplasm	other	-2.232	0.005
H3.3 histone A	P84245	H3-3A/H3-3B	Nucleus	other	-2.252	0.038
Receptor for activated C kinase 1	P63245	RACK1	Cytoplasm	enzyme	-2.326	0.009
Threonyl-tRNA synthetase 1	Q5XHY5	TARS1	Nucleus	enzyme	-2.661	0.012

The intensities of proteins were calculated using the Proteome Discoverer software, and the annotation of protein cellular localisation was performed by the Ingenuity Pathway Analysis Log2FC: Log2 (fold change). ES, extracellular space; PM, plasma membrane; TR, transcription regulator; Tm Rc, transmembrane receptor

## Discussion

Numerous studies related to KRG safety only examined its effects when administered for 8–12 wk. Recently, a large-scale clinical trial involving a 6 mo administration of KRG was performed [4]. However, more long-term studies on safety have yet to be conducted because clinical trials involving people are limited by cost and time. In this study, the toxicity of KRG measured during 4, 8, and 12 mo of repeated oral administration to rats was evaluated; analysing the mortality and clinical characteristics revealed no marked difference among all treatment and control groups. Most haematological and blood chemistry parameters showed no significant difference between the control and KRG-treated group. Although significant differences were found in some indicators, all were minor changes within the normal range and had no clinical significance [7]. In addition, there were no significant histopathological changes in the evaluated organs, even though spontaneous or ageing-related findings were observed in the male and female administration groups. Therefore, this study’s results indicate that oral administration of KRG did not produce any significant toxic effects.

In addition, this study attempted to confirm the changes in the ratio of immune cells in the thymus and spleen after administering KRG for 12 mo. Results show that the ratio of NK cells, T cells, and DC cells significantly increased in the male thymus. The ratio of T cells also significantly increased in the female thymus, and the NK cell activity increased in the male spleen. In a recent study, age-dependent alteration of immune cell subtypes in mice, such as a decrease of NK and CD4 T cells, was identified through single-cell profiling of the immune system. Therefore, the results obtained by this study could be evidence that long-term KRG intake has potent anti-immunosenescence effects [8]. A 12-mo-old mouse is considered middle-aged (10–14 mo), equivalent to 38–47 year in humans. Middle age refers to a phase during which senescent changes can be detected in some biomarkers, and senescence in younger adults can be detected [9]. Presumably, long-term administration of KRG can affect innate, mediated, and acquired immunity in middle-aged rats. Although changes in the immune cells in males compared to females were deemed significant, comparing them at an age range with evident age-related changes might be able to provide more explicit research implications.

Meanwhile, proteomic analysis was performed on blood obtained after 12 mo. The 11-plex TMT proteomic analysis of the sera revealed differentially expressed proteins (DEPs) based on biological function, and the analysis showed most proteins were defence/immunity and binding proteins. A more detailed bioinformatic interpretation showed that viral infection incidences decreased in male and female rat groups when administered with KRG for 12 mo. In male rats, concomitant infections caused by RNA viruses and Human Immunodeficiency Virus (HIV) decreased. The analysis confirmed that CYCS (HGNC:4367), RAP1B (HGNC:9857), MANaA1, and ITGB1 (HGNC:6153) decreased, and GM2A (HGNC:4367) increased, all of which have common functions involved with RNA viruses and HIV infection.

RAP1 is a member of the Ras superfamily of monomeric GTPases closely related to Ras, which regulates several signal pathways involved in cell formation, immune response, polarity, and apoptosis and has two isoforms (RAP1A and RAP1B). Current data suggest that RAP1B is overexpressed in many tumours [10]. In addition, it was reported that the hepatitis B virus (HBV) downregulated microRNA-101 (mir-101) by upregulating RAP1B, which promoted the proliferation and migration of HCC [11]. Meanwhile, ITGB1, also known as CD29, is a cell surface receptor that serves as an important host factor for rabies virus (RABV) infection. The presence of RABV drastically decreased after ITGB1 interfered with RNA knockdown and moderately increased after ITGB1 was overexpressed in cells [12]. Other studies also reported that the downregulation of ITGB1 decreased the number of early infections with human cytomegalovirus, Ebola virus, parvovirus, and reovirus [12]. The GM2 ganglioside activator, also known as GM2A, is a lipid transfer protein that stimulates the enzymatic processing of gangliosides and T-cell activation through lipid presentation [13]. Several proteins, including GM2A, are underexpressed in CD4 lymphoblasts after HIV inoculation [14]. In addition, genome-scale siRNAs screening showed GM2A acted as a potential restricting factor for HIV [15]. These reports suggested that their hypoexpression could be associated with a high virus replication rate in human cells. Transgelin (TAGLN) family members, which contains three isotopes, have been identified as actin crosslinking/gelling proteins. Specifically, TAGLN2 (transgelin2 or SM22; HGNC:1154), a putative actin crosslinking/gelling protein, plays an important role in the early stages of HIV-1 infection, as observed in a siRNA analysis. In addition, some reports indicated that TAGLN2 was overexpressed in gastric cancers and hepatocellular carcinomas [16, 17]. All proteins concerning HIV-1 and RNA virus infections and cell infections in the female rats were lower. It was also reported that mutant ATP5F1B (unspecified knockdown) decreased the replication of HSV-1 [18]. In addition, the interference of human ATP5F1B (HGNC:830) and CCT2 (HGNC:1615) mRNA by siRNA decreases the productive infection of HeLa P4/R5 cells of HIV-1 HXB2 [15]. Specifically, a fragment (169–263) from the DHX9 protein increases the infection of MT-2 cells in a cell culture infected with HIV-1 [19]. It also reported that human PSMA5 mRNA interference by siRNA decreases HEK293T cells’ infection caused by HIV-1.

## Conclusion

This study’s results suggested that long-term KRG administration for 12 mo is considered safe as no notable toxicological changes occurred. The study confirmed that the immune cell counts increased, and the proteins’ changes were related to reducing infections.

## Electronic supplementary material

Below is the link to the electronic supplementary material.

Supplementary Material 1

Supplementary Material 2

Supplementary Material 3

Supplementary Material 4

Supplementary Material 5

Supplementary Material 6

## Data Availability

The datasets and mass spectrometry proteomics data generated and analysed during the current study have been deposited to the ProteomeXchange Consortium via the PRIDE [20] partner repository. The datasets used during the current study are available from the corresponding author on reasonable request [dataset identifier PXD032036].

## Abbreviations

ABC: Ammonium bicarbonate.
A/G: Albumin-Globulin ratio.
AGC: Automatic Gain Control.
ALB: Albumin.
ALP: Alkaline phosphatase.
ALT: Alanine aminotransferase.
AST: Aspartate aminotransferase.
BCA: Bicinchoninic acid.
BUN: Blood urea nitrogen.
CHO: Total cholesterol.
CID: Collision-Induced Dissociation.
CPK: Creatine phosphokinase.
CRE: Creatinine.
DEPs: Differentially Expressed Proteins.
DTT: Dithiothreitol.
EDTA: Ethylenediaminetetraacetic acid.
FDR: False Discovery Rate.
GLU: Glucose.
H & E: Haematoxylin and Eosin.
Hb: Haemoglobin.
HCD: Higher-energy Collision Dissociation.
Hct: Haematocrit.
IAA: Iodoacetamide.
IPA: Ingenuity Pathway Analysis.
KRG: Korean Red Ginseng.
MCH: Mean Corpuscular Haemoglobin.
MCHC: Mean Corpuscular Haemoglobin Concentration.
MCV: Mean Corpuscular Volume.
NCE: Normalised Collision Energy.
PLT: Platelet.
RABV: Rabies virus.
RBC: Red Blood Cell.
RPLC: Reverse-Phase Liquid Chromatography.
SPS: Synchronous Precursor Selection.
TAGLN: Transgelin.
TG: Triglyceride.
TMT: Tandem Mass Tag.
TP: Total Protein.
UPLC: Ultra-Performance Liquid Chromatography.
WBC: White Blood Cell.
