# Supplementary material for: Long-term evaluation of safety and biological effects of Korean Red Ginseng (Panax Ginseng): a long-term in vivo study
Source: BMC Complement Med Ther. 2022 Nov 4;22:284. doi: 10.1186/s12906-022-03736-5 (PMC9635099; doi:10.1186/s12906-022-03736-5)
Supplement: Supplementary file 1 — Supplementary Material 1 [file 12906_2022_3736_MOESM1_ESM.pdf]

Data S1. Serum protein list in male rats administered with KRG for 12 mo.

Description	Accession	Gene	Log2(Fold change)	p-value
C4b-binding protein alpha chain	Q63514	C4bpa	-0.88	0.00307
Ac1873	Q7TQ70	Fga	0.59	0.05605
Collagen alpha-1(I) chain	P02454	Col1a1	0.43	0.18838
Thymosin beta-4	P62329	Tmsb4x	-0.91	0.04727
Serum albumin	P02770	Alb	0.02	0.94601
Protein S (Alpha), isoform CRA_b	M0R5R0	Pros1	-0.34	0.21570
Collagen alpha-1(III) chain	P13941	Col3a1	0.41	0.29700
Angiopoietin-like 3	F7FHP0	Angptl3	0.15	0.56103
Apolipoprotein B-100	F1M6Z1	Apob	-0.60	0.03614
C4b-binding protein beta chain	A0A5C5	C4bpb	-1.33	0.00213
Angiopoietin-like 8	D3ZMI0	Angptl8	0.20	0.47535
Serum amyloid A protein	Q5M878	Saa4	0.31	0.35325
Collagen, type I, alpha 2	A0A0G2KAN1	NEWGENE_(	-0.04	0.89797
Colony stimulating factor 1 receptor	A0A0G2KBC4	Csf1r	-0.19	0.33038
Apolipoprotein M	P14630	Apom	0.01	0.96967
Protein Z-dependent protease inhibitor	Q62975	Serpina10	-1.18	0.00148
Cathelicidin antimicrobialptide	G3V8S9	Camp	0.03	0.92515
Protein Z, vitamin K-dependent plasma glyco	G3V8K8	Proz	-0.03	0.90647
Thyroxine-binding globulin	A0A140TAB0	Serpina7	-0.38	0.32197
Vasodilator-stimulated phosphoprotein	A0A0G2K9C0	Vasp	-0.55	0.09236
Inter alpha-trypsin inhibitor, heavy chain 4	Q5EBC0	Itih4	-0.96	0.00518
Apolipoprotein E	P02650	Apoe	0.50	0.06085
Carboxypeptidase	Q6AYS3	Ctsa	0.27	0.13432
Apolipoprotein C-I	P19939	Apoc1	0.26	0.51916
Maltase-glucoamylase	D3ZTX4	Mgam	-0.17	0.70403
Proprotein convertase subtilisin/kexin type 9	P59996	Pcsk9	0.30	0.47512
Apolipoprotein C-III	A0A0G2K8Q1	Apoc3	0.26	0.49920
CD44 antigen	D3ZGF1	Cd44	-0.05	0.79087
HGF activator	Q5EBA7	Hgfac	-0.50	0.01233
Receptor protein-tyrosine kinase	G3V6K6	Egfr	0.12	0.48767
Ig-like domain-containing protein	F1LWD0		-1.07	0.00055
Lipopolysaccharide-binding protein	Q63313	Lbp	-0.47	0.12686
Filamin A	C0JPT7	Flna	-0.75	0.01570
Alpha-2-macroglobulin	P06238	A2m	0.24	0.67080
Coagulation factor X	Q63207	F10	-0.32	0.15988
Apolipoprotein C-IV	P55797	Apoc4	0.23	0.62243
Glycosylphosphatidylinositol specific phosphc	G3V8B1	Gpld1	-0.03	0.93208
Alpha-2-macroglobulin-like 1	D3ZS19	A2ml1	-0.33	0.41314
Tropomyosin alpha-3 chain	Q63610	Tpm3	-0.82	0.03376
FYN-binding protein 1	D3ZIE4	Fyb1	-0.88	0.03704
Coactosin-like protein	B0BNA5	Cotl1	-0.44	0.24536
Apolipoprotein C-II (Predicted)	G3V8D4	Apoc2	0.58	0.15700
Serine protease inhibitor Kazal-type 1-like	P09656	Spink1l	0.27	0.34579
Dentin sialophosphoprotein	Q62598	Dspp	0.34	0.11548
Fibrinogen gamma chain	P02680	Fgg	-0.29	0.26933
Tubulin beta chain	G3V7C6	Tubb4b	-0.41	0.32448
Serum amyloid P-component	P23680	Apcs	-0.16	0.48672
Lipoprotein lipase	Q06000	Lpl	-0.10	0.68125
Ig-like domain-containing protein	F1MAE7		-0.08	0.86157
PDZ and LIM domain protein 1	P52944	Pdlim1	-0.41	0.30860
All-trans-retinol dehydrogenase [NAD(+)] ADI	G3V7J9	Adh7	-0.54	0.02411
Complement C7	A0A0G2K7X7	C7	0.13	0.55203
Apolipoprotein A-I	P04639	Apoa1	0.03	0.93177

Description	Accession	Gene	Log2(Fold change)	p-value
Fibrinogen-like protein 1	Q5M8C6	Fgl1	-0.70	0.09694
Serotransferrin	P12346	Tf	0.51	0.28253
Peroxiredoxin-5, mitochondrial	Q9R063	Prdx5	-0.51	0.04549
Extracellular superoxide dismutase [Cu-Zn]	Q08420	Sod3	0.22	0.24373
Dipeptidase 2	Q5M872	Dpep2	0.40	0.03835
Heparin cofactor 2	A0A0G2K8K3	Serpind1	-0.79	0.02578
Protein AMBP	Q64240	Ambp	-0.25	0.43008
Histone H2A	D4AEC0	H2afv	-2.29	0.00255
Inter-alpha-trypsin inhibitor heavy chain 2	D3ZFH5	Itih2	-0.34	0.16077
Glyceraldehyde-3-phosphate dehydrogenase	P04797	Gapdh	-0.20	0.48676
Tropomyosin alpha-4 chain	P09495	Tpm4	-1.15	0.00340
Apolipoprotein A-II	P04638	Apoa2	0.73	0.05777
Ig gamma-2C chain C region	P20762		0.06	0.91536
Serine protease inhibitor A3L	P05544	Serpina3l	-0.25	0.29214
Pro-neuropeptide Y	P07808	Npy	0.77	0.08612
Alpha-2-HS-glycoprotein	P24090	Ahsg	0.20	0.31403
Regulator of G-protein signaling 18	Q4L0E8	Rgs18	-0.19	0.59507
C-type lectin domain family 3, member B	D3ZUU6	Clec3b	-0.25	0.26093
Prothrombin	G3V843	F2	-0.01	0.96037
Tubulin beta-2A chain	P85108	Tubb2a	-0.32	0.38589
RCG28243	D3ZQV0	Prss3	-0.85	0.00222
Afamin	G3V9R9	Afm	0.29	0.31562
Platelet-activating factor acetylhydrolase	Q5M7T7	Pla2g7	-0.29	0.33547
Ig-like domain-containing protein	M0RDF2		-0.76	0.00924
CD248 antigen, endosialin (Predicted)	D3ZN06	Cd248	0.59	0.29662
Inter-alpha trypsin inhibitor, heavy chain 1	B2RYM3	Itih1	-0.39	0.08147
Fibronectin	F1LST1	Fn1	-0.57	0.04100
Transcription factor-binding to IGHM enhanc	D3ZAW6	Tfe3	0.87	0.01528
Serum paraoxonase/lactonase 3	Q68FP2	Pon3	-0.18	0.66963
Major urinary protein 4	F8WFF8	Mup4	-0.46	0.18891
Ig gamma-2B chain C region	P20761	Igh-1a	0.40	0.37887
Fc fragment of IgG-binding protein	D3ZJF8	Fcgbp	0.68	0.02357
Cd300 molecule-like family member E	F1M9Z5	Cd300le	0.44	0.12490
Serum paraoxonase/arylesterase 1	P55159	Pon1	-0.76	0.04809
Histidine-rich glycoprotein	A0A0G2K3G0	Hrg	-0.82	0.04971
Alpha-aminoadipic semialdehyde dehydroger	Q64057	Aldh7a1	-0.33	0.59188
Lecithin cholesterol acyltransferase	O35849	Lcat	0.00	0.99425
Biotinidase	A0A140TAI2	Btd	-0.17	0.39505
Alpha-1-macroglobulin	Q63041	A1m	0.05	0.84145
RCG49849	D3ZAE6	Vasn	-0.35	0.10738
Immunoglobulin heavy constant mu	F1LN61	Ighm	-0.68	0.14390
Uncharacterized protein	F1LTJ5		0.56	0.01024
Ig-like domain-containing protein	A0A0G2JXF0		0.53	0.18601
Complement C3	M0RBF1	C3	-0.35	0.04062
Profilin-1	P62963	Pfn1	-0.84	0.02113
Similar to RIKEN cDNA 1300017J02	A0A0G2K896	RGD131050	0.20	0.42580
L-selectin	F7EY63	Sell	0.46	0.13208
Keratin, type I cytoskeletal 10	Q6IFW6	Krt10	-0.04	0.91130
Rho GDP-dissociation inhibitor 1	Q5XI73	Arhgdia	-0.43	0.14342
Carboxylesterase 1C	P10959	Ces1c	-0.95	0.04250
Alpha-1B-glycoprotein	Q9EPH1	A1bg	-0.92	0.01612
Protein C, isoform CRA_b	F7FMY6	Proc	0.56	0.01977
Alpha-2 antiplasmin	Q80ZA3	Serpinf1	-0.26	0.20169
Ig-like domain-containing protein	F1LYQ4		-1.36	0.01429

Description	Accession	Gene	Log2(Fold change)	p-value
Carboxypeptidase Q	Q6IRK9	Cpq	0.09	0.75324
Growth hormone receptor	I1SRC4	Ghr	-0.56	0.08706
C-C motif chemokine 6	Q68FP3	Ccl6	0.37	0.09882
Deleted in malignant brain tumors 1 protein	Q8CIZ5	Dmbt1	-1.43	0.02474
Heterogeneous nuclear ribonucleoprotein U	Q6IMY8	Hnrnpu	-0.60	0.16036
Carbonic anhydrase 2	P27139	Ca2	0.65	0.03313
Beta-2-microglobulin	P07151	B2m	0.19	0.37757
Transgelin-2	Q5XFX0	Tagln2	-1.27	0.00778
RCG62531, isoform CRA_g	A0A140TAF0	Tpm3	0.16	0.71663
Ceruloplasmin	G3V7K3	Cp	-1.15	0.00172
Urinary protein 1-like	D3ZIF6	LOC100360(	-0.34	0.52442
Serpin A11	Q7TPA5	Serpina11	-0.47	0.08802
Laminin subunit alpha 2	F1M614	Lama2	0.48	0.15616
Zinc finger and BTB domain containing 7a	G3V8P6	Zbtb7a	-0.70	0.02198
Collagen type XVIII alpha 1 chain	F1LR02	Col18a1	-0.36	0.16603
Insulin-like growth factor-binding protein 3	P15473	Igfbp3	0.24	0.35247
Protein disulfide-isomerase	P04785	P4hb	-0.39	0.03330
Keratin, type I cytoskeletal 42	Q6IFU7	Krt42	0.07	0.88590
Plasma protease C1 inhibitor	Q6P734	Serping1	-0.66	0.01944
Vascular endothelial growth factor receptor 2	O08775	Kdr	0.18	0.26857
Retinoic acid receptor responder (Tazarotene	Q5BK77	Rarres2	0.10	0.76699
RCG25684, isoform CRA_a	Q6AXW2	Tmod3	0.15	0.73376
Alpha glucosidase 2 alpha neutral subunit (Pr	D3ZAN3	Ganab	0.01	0.97330
Coagulation factor XIII A chain	G3V811	F13a1	0.59	0.07199
Carboxypeptidase N subunit 2	F1LQT4	Cpn2	-1.30	0.00350
Complement component C8 beta chain	P55314	C8b	-1.74	0.01274
Corticosteroid-binding globulin	P31211	Serpina6	-0.84	0.03309
C-reactive protein	P48199	Crp	0.30	0.14617
Peptidoglycan recognition protein 2	M0R485	Pglyrp2	-0.16	0.57016
Alpha-1-antiproteinase	A0A0G2JZ73	Serpina1	-0.34	0.41982
Vinculin	A0A0G2K8V2	Vcl	-0.61	0.01054
Ig-like domain-containing protein	A0A0G2JV42		0.48	0.17894
Keratin, type II cytoskeletal 1	Q6IMF3	Krt1	0.68	0.07643
Ig-like domain-containing protein	D3ZFF8		0.74	0.14352
Cytochrome c, somatic	P62898	Cycs	-0.81	0.02002
Ab2-183	Q7TP53	Il2rg	0.12	0.76815
Ig-like domain-containing protein	M0RBK4		0.86	0.09762
Apolipoprotein D	M0R4S2	Apod	-1.47	0.07673
Lumican	P51886	Lum	-0.33	0.42297
T-kininogen 1	P01048	Map1	-0.08	0.84675
Annexin A2	Q07936	Anxa2	0.46	0.30358
Peptidase inhibitor 16	D3ZGM7	Pi16	0.35	0.27708
Complement factor H-related protein B	A0A0G2JYC4	LOC100361(	0.18	0.50340
Ig-like domain-containing protein	A0A0G2K0N6		0.80	0.06468
Coagulation factor XI	A0A0G2K4I9	F11	-0.47	0.18839
Tsukushin	Q6QMY6	Tsku	1.13	0.01029
Cofilin-1	P45592	Cfl1	-0.86	0.00482
Tubulin alpha-4A chain	Q5XIF6	Tuba4a	-1.65	0.00041
Integrin beta-3	Q8R2H2	Itgb3	-0.48	0.11425
Proteasome subunit alpha type	A0A0G2K0W9	Psma7	0.08	0.72541
Elongation factor 1-alpha	M0R757	LOC100360(	-0.31	0.32235
Complement C5	A0A096P6L9	C5	-0.58	0.33662
Inducible T-cell co-stimulator ligand	F1LVL2	Icoslg	-0.38	0.03529
Apolipoprotein N	Q5M890	Apon	-0.16	0.70267

Description	Accession	Gene	Log2(Fold change)	p-value
Ig-like domain-containing protein	D3ZZ08		0.42	0.24752
Alpha-tropomyosin 3	Q63607	Tpm1	-0.24	0.51330
Apolipoprotein A-IV	P02651	Apoa4	0.09	0.73788
ADF-H domain-containing protein	D4A315		0.50	0.20561
Leukemia inhibitory factor receptor	G3V7K2	Lifr	-0.40	0.27361
Kininogen-1	P08934	Kng1	-0.54	0.01269
RCG55135, isoform CRA_b	G3V852	Tln1	-0.69	0.15749
Serpin family F member 2	F7FHF3	Serpinf2	0.34	0.10357
Pyruvate kinase PKM	P11980	Pkm	-0.54	0.20701
Mannan-binding lectin serineptidase 2	A2VCV7	Masp2	-0.45	0.24342
Alpha-2-glycoprotein 1, zinc	Q3B8R6	Azgp1	-0.12	0.47179
Heart development protein with EGF-like don	F1M9I4	Heg1	0.68	0.01532
Serine protease inhibitor A3K	P05545	Serpina3k	-0.34	0.13690
Complement C1q subcomponent subunit C	P31722	C1qc	0.28	0.29346
Ig-like domain-containing protein	M0R8G6		0.62	0.13757
Fibrinogen beta chain	P14480	Fgb	-0.19	0.74914
Secreted phosphoprotein 24	A0A0G2K9X1	Spp2	0.11	0.75695
Ig-like domain-containing protein	F1LZH0		0.50	0.32595
Ig-like domain-containing protein	D3ZWC1		-0.22	0.49276
Ig-like domain-containing protein	A0A0G2K3L1		0.36	0.51848
Hyaluronidase	F1M963	Hyal1	0.01	0.98230
Pleckstrin	A0A0G2K393	Plek	-1.12	0.00349
Neural cell adhesion molecule 1	F1LNY3	Ncam1	-0.06	0.85841
ATP synthase subunit beta, mitochondrial	P10719	Atp5f1b	-0.53	0.44041
cGMP-specific 3',5'-cyclic phosphodiesterase	O54735	Pde5a	-0.46	0.34563
Thioredoxin domain-containing protein	A0A0G2K3Z9		0.30	0.25146
Urinary protein 2	P81828		1.03	0.01732
Ficolin (Collagen/fibrinogen domain containir	Q5M8B4	Fcna	0.25	0.26284
Protein disulfide-isomerase	A0A0H2UHM5	Pdia3	-0.30	0.23050
Hemoglobin subunit beta-1	P02091	Hbb	0.19	0.57718
Adenylyl cyclase-associated protein 1	Q08163	Cap1	-1.13	0.01137
Prosaposin	F7EPE0	Psap	0.22	0.39686
Murinoglobulin-1	Q03626	Mug1	-0.52	0.12488
Dipeptidylptidase 4	A0A0G2JTX5	Dpp4	0.21	0.27769
Lymphocyte cytosolic protein 1	Q5XI38	Lcp1	0.15	0.59440
Glycoprotein Ib platelet subunit alpha	D3ZQU7	Gp1ba	-0.34	0.34760
Similar to Ig variable region, light chain	F1M7I8	RGD156561	1.07	0.11887
Extracellular link domain-containing 1 (Predic	D3ZD19	Lyve1	0.43	0.05983
Contactin-1	Q63198	Cntn1	0.09	0.69590
Heat shock 70 kDa protein 1A	P0DMW0	Hspa1a	-0.61	0.19903
Superoxide dismutase [Cu-Zn]	P07632	Sod1	0.06	0.81869
Junction plakoglobin	Q6P0K8	Jup	0.46	0.21936
Mannan-binding lectin serine protease 2	A0A0G2K392	Masp2	-1.10	0.20222
Complement factor I	A0A0G2K135	Cfi	0.12	0.40217
Multimerin 1	D4A3E0	Mmrn1	0.17	0.45035
Similar to RIKEN cDNA 1300017J02	E9PST1	RGD131050	-0.81	0.02235
Ig-like domain-containing protein	M0R8B5		0.75	0.11375
Ig-like domain-containing protein	F1M4R1		0.50	0.16600
Fibrinogen-like 2	G3V7P2	Fgl2	0.49	0.05542
Nucleoside diphosphate kinase B	P19804	Nme2	-0.76	0.02619
Osteopontin	P08721	Spp1	1.14	0.00616
Keratinocyte differentiation-associated proteii	P85411	Krtdap	0.16	0.68048
Complement C1s subcomponent	G3V7L3	C1s	-0.91	0.04919
Coagulation factor V	A0A0G2K3W2	F5	-0.57	0.17478

Description	Accession	Gene	Log2(Fold change)	p-value
Ig-like domain-containing protein	D3ZAB3		1.18	0.04608
F-actin-capping protein subunit alpha-1	B2GUZ5	Capza1	-0.56	0.12470
Plasma kallikrein	P14272	Klkb1	-0.13	0.43585
Ceruloplasmin	P13635	Cp	-0.05	0.87354
Uncharacterized protein	F1LTN6		0.52	0.45355
Clusterin	G3V836	Clu	0.14	0.35752
Afamin	P36953	Afm	0.56	0.30910
Leukocyte cell-derived chemotaxin 2	D4A526	Lect2	0.35	0.27329
alpha-1,2-Mannosidase	A0A0G2JW29	Man1a1	-0.73	0.02644
Keratin, type II cytoskeletal 2 epidermal	A0A0G2JWX4	Krt2	0.25	0.62857
Apolipoprotein C-I-like	M0R547	LOC100910	-0.21	0.56378
Polymeric immunoglobulin receptor	A0A0G2K5U5	Pigr	-0.36	0.67874
Coagulation factor IX	P16296	F9	-0.83	0.05127
RCG46174	G3V7P5	RGD156461	0.30	0.12376
T-kininogen 2	P08932		-0.70	0.27260
Similar to BC049975 protein	F1M8F5	LOC100909	-0.71	0.03894
Na(+)/H(+) exchange regulatory cofactor NHI	Q9JJ19	Slc9a3r1	-0.81	0.11164
Desmoplakin	F1LMV6	Dsp	0.35	0.19846
Protein S100-A9	A0A0H2UHJ1	S100a9	-0.52	0.34814
Sulfhydryl oxidase 1	Q6IUU3	Qsox1	0.19	0.50359
Ig-like domain-containing protein	M0RDL2		0.54	0.26635
Keratin 71	D3ZXB7	Krt71	-0.35	0.28556
Ig-like domain-containing protein	A0A0G2JY98		0.45	0.40838
Aspartate aminotransferase, cytoplasmic	P13221	Got1	-0.39	0.15165
Complement C4	P08649	C4	-0.18	0.47880
Plasminogen activator inhibitor 2 type A	P29524	Serpinb2	0.28	0.15934
Urinary protein 3-like	M0R7P3	LOC100912	1.00	0.00102
Alpha-amylase	E9PSQ1	Amy1a	-0.31	0.28047
Glutathioneroxidase 1	P04041	Gpx1	0.13	0.72257
Proteasome subunit beta type-6	P28073	Psmb6	0.59	0.04513
Carbonic anhydrase 3	P14141	Ca3	-1.32	0.14790
Out at first protein homolog	Q6AYE5	Oaf	0.57	0.03722
Galectin	B4F7A3	Lgalsl	0.08	0.84343
Complement C8 gamma chain	D3ZPI8	C8g	-0.60	0.00254
Ig-like domain-containing protein	A0A0G2JX36		0.14	0.79459
Ig-like domain-containing protein	M0R9U2		0.58	0.10508
Transforming growth factor, beta-induced	D4A8G5	Tgfbi	-0.04	0.90870
Metalloproteinase inhibitor 2	P30121	Timp2	0.00	0.98789
Keratin, type II cytoskeletal 6A	Q4FZU2	Krt6a	0.46	0.28545
Platelet factor 4	P06765	Pf4	0.71	0.06185
Complement C1q subcomponent subunit A	P31720	C1qa	-0.02	0.92771
Ig-like domain-containing protein	F1M5L5		0.12	0.68848
Attractin	Q99J86	Atrn	0.09	0.80264
Plexin domain containing 2 (Predicted)	B5DEZ8	Plxdc2	-0.08	0.80677
Protein tyrosine phosphatase, receptor type,	F1LP13	Ptprg	-0.05	0.85746
Tyrosine-protein kinase Mer	P57097	Mertk	0.27	0.17763
Uncharacterized protein	F1LWS4	Cfhr2	-0.31	0.32226
Immunoglobulin heavy constant mu	F1LPR6	Ighm	-0.76	0.26938
Rho GDP dissociation inhibitor beta	Q5M860	Arhgdib	-0.03	0.91411
Lysyl oxidase-like 1	Q5FWS5	Loxl1	0.65	0.04348
Fetuin-B	Q9QX79	Fetub	-0.38	0.26904
CD163 antigen (Predicted)	D3Z9U2	Cd163	0.68	0.00427
Dipeptidylptidase 1	P80067	Ctsc	0.21	0.40421
Ig-like domain-containing protein	A0A0G2K6T8		-0.33	0.41842

Description	Accession	Gene	Log2(Fold change)	p-value
Adiponectin a	G3V7N9	C1qb	0.13	0.61269
RCG61833	F1LQS6	Xdh	-0.26	0.13413
Ig-like domain-containing protein	F1LZ11		0.81	0.14326
Resistin-like gamma	G3V686	Retnlg	-0.27	0.40404
Synaptic vesicle membrane protein VAT-1 ho	Q3MIE4	Vat1	0.23	0.29235
Lipase	D3ZUQ1	Lipo1	-0.45	0.20474
Carboxypeptidase N catalytic chain	Q9EQV8	Cpn1	-0.46	0.24065
Ig kappa chain V region S211	P01681		0.10	0.74519
Complement C4B (Chido blood group)	Q6MG90	C4b	0.03	0.91275
CD55 molecule (Cromer blood group)	A0A0G2QC50	Cd55	-0.10	0.70450
Histone H4	P62804	H4c2	-0.61	0.52883
Cadherin 13	Q8R490	Cdh13	0.32	0.26991
Di-N-acetylchitobiase	Q01460	Ctbs	-0.44	0.15741
C1r protein	B5DEH7	C1r	0.32	0.26474
Extracellular matrix protein 1	Q62894	Ecm1	0.25	0.15788
Hemoglobin subunit alpha-1/2	P01946	Hba1	-0.50	0.17517
Alpha-1-inhibitor 3	P14046	A1i3	-0.30	0.44368
Moesin	A0A096MK30	Msn	-0.59	0.06857
Insulin-like growth factor 2 receptor	G3V824	Igf2r	0.25	0.26890
Immunoglobulin joining chain	G3V6G1	Jchain	0.67	0.06763
Angiotensin-converting enzyme (Fragment)	A0A0A0MXV4	Ace	-0.03	0.92996
Serine protease inhibitor A3M	F1LR92	Serpina3m	-0.06	0.87505
Tubulin beta chain	M0R8B6	Tubb1	-1.22	0.01055
Inter-alpha-trypsin inhibitor heavy chain H3	D3ZBS2	Itih3	-0.42	0.51900
Uncharacterized protein	G3V9J1		-0.28	0.38473
Glutathioneroxidase	A0A0G2K531	Gpx3	-1.02	0.02177
Ig-like domain-containing protein	F1M1R0	LOC690813	1.04	0.02631
Mannose-binding protein C	P08661	Mbl2	0.48	0.03039
Ig-like domain-containing protein	D3ZHM9		0.82	0.17594
Mannose-binding protein A	P19999	Mbl1	-0.86	0.25287
Ras-related protein Rap-1b	Q62636	Rap1b	-1.67	0.00098
Hemopexin	P20059	Hpx	-0.43	0.12862
Thrombospondin 1	M0R979	Thbs1	-0.07	0.75957
Antileukoproteinase-like 2	G3V9C3	Slpil2	0.30	0.30297
Serine (Or cysteine)ptidase inhibitor, clade C (	Q5M7T5	Serpinc1	-0.24	0.53213
Keratin, type II cytoskeletal 5	Q6P6Q2	Krt5	0.45	0.32645
Antigen p97 (Melanoma associated) identifie	D4ADK7	Meltf	0.70	0.44691
Ig-like domain-containing protein	D3ZQR5		-0.10	0.72575
Guanylate cyclase activator 2B	P70668	Guca2b	0.66	0.01342
Uncharacterized protein	M0R5J4		-0.75	0.01472
Keratin, type II cytoskeletal 8	Q10758	Krt8	0.24	0.68046
Protein S100-A8	P50115	S100a8	-0.77	0.40089
Complement C8 alpha chain	D3ZWD6	C8a	-0.17	0.28417
Ig-like domain-containing protein	D3ZEP5		1.20	0.02330
Serine (Or cysteine) proteinase inhibitor, clad	Q5M8C3	Serpina4	-0.48	0.15372
Murinoglobulin-2	Q6IE52	Mug2	-0.32	0.26445
Cysteine and glycine-rich protein 1	P47875	Csrp1	-0.98	0.02177
Cathepsin B	Q6IN22	Ctsb	-0.22	0.55568
Protein S100-A4	P05942	S100a4	-0.59	0.03728
Alpha-1-acid glycoprotein	P02764	Orm1	-0.52	0.30990
Ig-like domain-containing protein	A0A0G2K7S9		0.45	0.38231
Transaldolase	Q9EQS0	Taldo1	0.14	0.51300
Insulin-like growth factor-binding protein 4	P21744	Igfbp4	0.61	0.14442
Phospholipase A2, membrane associated	P14423	Pla2g2a	0.04	0.90770

Description	Accession	Gene	Log2(Fold change)	p-value
Cystatin-C	P14841	Cst3	0.52	0.03294
Keratin, type II cytoskeletal 75	Q6IG05	Krt75	0.02	0.98403
Plasminogen	Q01177	Plg	-0.29	0.14134
Similar to Vanin-3 (Predicted)	D4A183	Vnn3	0.22	0.53503
Zyxin	D4A7U1	Zyx	-0.21	0.61784
Trypsin	P00761		-0.29	0.17726
Uteroglobin	P17559	Scgb1a1	0.11	0.75977
Carboxylic ester hydrolase	D3ZGK7	Ces1c	-0.87	0.02698
Triggering receptor-expressed on myeloid cel	D3ZYT6	Treml1	-0.47	0.27941
Ig-like domain-containing protein	M0R816		0.92	0.11777
Metalloproteinase inhibitor 3	Q4V8L0	Timp3	-0.05	0.90362
Selenoprotein P	P25236	Selenop	0.29	0.32152
Tubulin alpha-1B chain	Q6P9V9	Tuba1b	-0.32	0.31428
Phosphoglycerate kinase 1	P16617	Pgk1	-0.88	0.00241
Insulin-like growth factor binding protein, aci	F1LRE2	Igfals	-0.58	0.04705
Ig-like domain-containing protein	A0A0G2JXB7		1.00	0.00518
Complement factor H	G3V9R2	Cfh	-0.37	0.48469
Insulin-like growth factor I	A0A0G2JX40	Igf1	0.33	0.17597
Plexin B2	D3ZQ57	Plxnb2	0.42	0.14442
Adiponectin, C1Q and collagen domain-conta	A0A0G2K845	Adipoq	-0.67	0.16595
Proteoglycan 4	F1LRA5	Prg4	-0.26	0.47360
Endoplasmic reticulum chaperone BiP	P06761	Hspa5	-0.23	0.53788
Complement component C9	Q62930	C9	0.14	0.65664
Pulmonary surfactant-associated protein D	P35248	Sftpd	0.68	0.09598
Lysosome-associated membrane glycoprotein	P17046	Lamp2	-0.18	0.39905
Chromogranin-A	P10354	Chga	-0.35	0.53888
Hemoglobin subunit beta-2	P11517		-0.46	0.21167
Ig-like domain-containing protein	D3ZMS7		0.67	0.10063
CXC chemokine RTCK1	Q99ME0	Ppbp	0.12	0.71243
Transferrin receptor protein 1	G3V679	Tfrc	-0.25	0.48716
Proteasome subunit beta type-7	Q9JHW0	Psmb7	0.64	0.03235
GM2 ganglioside activator	Q6IN37	Gm2a	0.77	0.02312
Ubiquitin-60S ribosomal protein L40	P62986	Uba52	0.22	0.52532
Complement component factor h-like 1	Q5I0M3	Cfhr1	0.36	0.17456
RCG22683, isoform CRA_a	M0R8A3	Gp6	-0.61	0.02677
4F2 cell-surface antigen heavy chain	Q794F9	Slc3a2	-0.31	0.16900
Immunoglobulin heavy constant mu	F1LM30	Ighm	0.42	0.16222
Calponin (Fragment)	D3ZRX9	Cnn2	-1.39	0.01733
Complement factor properdin	B0BNN4	Cfp	0.46	0.04338
Complement component C6	Q811M5	C6	0.79	0.09431
Cystatin E/M	Q8VHC1	Cst6	0.54	0.01293
Major urinary protein	P02761		-0.10	0.79948
Ig-like domain-containing protein	M0RA79		0.10	0.84801
Angiotensinogen	P01015	Agt	-0.23	0.34768
Ferritin heavy chain	P19132	Fth1	-0.38	0.58230
Serum albumin	A0A0G2JSH5	Alb	-0.14	0.89333
Ig-like domain-containing protein	A0A0G2K5D2		0.41	0.18262
Keratin, type I cytoskeletal 17	Q6IFU8	Krt17	0.51	0.31716
Phospholipid transfer protein	E9PSP1	Pltp	-1.15	0.04719
Coagulation factor XIII B chain	F6Q1N1	F13b	0.07	0.72772
Integrin-linked protein kinase	Q99J82	Ilk	-0.40	0.31657
Ig-like domain-containing protein	F1M229		0.69	0.20498
Ig gamma-2A chain C region	P20760	Igg-2a	0.18	0.69577
Thrombospondin 1	A0A0G2JV24	Thbs1	0.31	0.30351

Description	Accession	Gene	Log2(Fold change)	p-value
Ig-like domain-containing protein	M0R7Q2		0.96	0.04702
Podocalyxin	A0A0G2K2L1	Podxl	0.13	0.62678
Protein-lysine 6-oxidase	P16636	Lox	0.53	0.50230
Complement factor B	G3V615	Cfb	-0.45	0.17435
Ig-like domain-containing protein	A0A0G2JUY3		0.12	0.88726
Apolipoprotein A-V	A0A0H2UHP7	Apoa5	0.25	0.33280
Actin, alpha skeletal muscle	P68136	Acta1	-2.26	0.01117
Retinol-binding protein 4	P04916	Rbp4	-0.52	0.03309
Ig-like domain-containing protein	A0A0G2K4K2		0.41	0.41490
Coagulation factor C homolog (Limulus polyp	B1H259	Coch	0.94	0.00793
Globin a1	Q62669	LOC1036948	-1.01	0.02030
Insulin-like growth factor-binding protein 2	P12843	Igfbp2	-0.14	0.72099
Interleukin-1 receptor accessory protein	F1M9B9	Il1rap	0.17	0.37815
Serine protease inhibitor A3N	A0A0H2UHI5	Serpina3n	-0.28	0.26745
Histone H2B	D4A817	Hist2h2be	0.44	0.68683
Fructose-bisphosphate aldolase A	P05065	Aldoa	-0.36	0.36606
Ig-like domain-containing protein	D3ZPL2		-0.09	0.87354
Similar to alpha-fetoprotein	F7FAY5	LOC360919	-0.23	0.22647
Glycoprotein 5, platelet	G3V9H9	NEWGENE_2	-0.41	0.19485
Fermitin family member 3	B2GVB9	Fermt3	-1.10	0.06095
Ig-like domain-containing protein	A0A0G2JZN1		0.48	0.28458
Ig kappa chain C region, A allele	P01836		0.86	0.09754
Keratin, type II cytoskeletal 4	A0A0G2K6P7	Krt4	0.28	0.28691
Histone H2A type 3	Q4FZT6		1.44	0.00781
Thioredoxin	R4GNK3	Txn1	-1.03	0.00305
Catalase	P04762	Cat	0.12	0.63857
von Willebrand factor	F1M957	Vwf	0.00	0.99325
Complement factor D	P32038	Cfd	-0.51	0.24901
Gamma-glutamyl hydrolase	Q62867	Ggh	-0.50	0.04251
Proteasome subunit alpha type-4	P21670	Psma4	0.55	0.06477
Keratin 16	Q6IFU9	Krt16	0.80	0.08575
Hyaluronan-binding protein 2	Q6L711	Habp2	0.48	0.02353
Serglycin	P04917	Srgn	-0.98	0.01172
Ig-like domain-containing protein	D4A4L6		0.20	0.74476
Peroxiredoxin-2	A0A0G2JSH9	Prdx2	0.30	0.30939
Ig-like domain-containing protein	A0A0G2JWF2		-0.08	0.88035
Ig-like domain-containing protein	A0A0G2JZV7		-0.03	0.94781
Myosin heavy chain 9-like 1	G3V6P7	Myh9	-0.94	0.07480
Elongation factor 2	P05197	Eef2	0.43	0.31064
Keratin, type II cytoskeletal 80	Q6IMF1	Krt80	0.40	0.17270
Insulin-like growth factor-binding protein 5	A0A0G2JVW1	Igfbp5	0.43	0.02298
Ig-like domain-containing protein	F1LTY5		0.36	0.41073
40S ribosomal protein SA	P38983	Rpsa	0.02	0.96977
RCG21066	D3ZJW6	rCG_21066	0.26	0.64413
Cathepsin L1	P07154	Ctsl	0.44	0.17660
Lysozyme C-1	P00697	Lyz1	0.56	0.09789
Complement C1r subcomponent-like protein	F1LP96	C1rl	-0.06	0.81666
Ig-like domain-containing protein	F1LXY6		-0.35	0.30715
Hepcidin	Q99MH3	Hamp	-0.34	0.35696
Actin, cytoplasmic 1	A0A0G2K3K2	Actb	-1.21	0.02020
Guanylin	P28902	Guca2a	0.70	0.06971
Integrin beta	A0A0G2JSK5	Itgb1	-0.85	0.00123
Heat shock cognate 71 kDa protein	P63018	Hspa8	-0.73	0.18611
Carboxylic ester hydrolase	G3V7J5	Ces2e	-0.49	0.45352

Description	Accession	Gene	Log2(Fold change)	p-value
RCG35022, isoform CRA_b	G3V6B7	Flt4	0.02	0.93757
Ig-like domain-containing protein	A0A0G2K8K8		0.45	0.27197
Apolipoprotein H	Q5I0M1	Apoh	-0.04	0.84293
Gelsolin	Q68FP1	Gsn	-1.13	0.05820
Proteasome subunit beta type-1	P18421	Psmb1	-0.02	0.94320
Caveolae-associated protein 2	Q66H98	Cavin2	-0.86	0.03973
CD5 antigen-like	Q4KM75	Cd5l	0.77	0.01981
Haptoglobin	P06866	Hp	-0.02	0.97247
Ig-like domain-containing protein	A0A0G2K1F0		0.51	0.20235
Ig-like domain-containing protein	M0R9S8		-0.27	0.46546
Coagulation factor XII	D3ZTE0	F12	-0.18	0.60065
Heterogeneous nuclear ribonucleoprotein C, i	A0A0G2JXW4	Hnrnpc	1.39	0.02643
Proteasome subunit alpha type-2	P17220	Psma2	-0.08	0.82649
Multiple inositol polyphosphate phosphatase	G3V7H2	Minpp1	-0.29	0.33823
Myeloperoxidase	A0A0G2K1A2	Mpo	0.51	0.19164
Ig lambda-2 chain C region	P20767		0.14	0.72784
Hephaestin-like 1	A0A0G2K8Q3	Hephl1	-0.29	0.30302
Vitronectin	Q3KR94	Vtn	-0.70	0.03072
14-3-3 protein zeta/delta	A0A0G2JV65	Ywhaz	-0.82	0.02681
Vascular cell adhesion protein 1	P29534	Vcam1	-0.53	0.09093
Fibulin-5	Q9WVH8	Fbln5	-0.42	0.35786
Adhesion G protein-coupled receptor E5	A0A0G2JSI4	Adgre5	-0.23	0.29531
Proteasome subunit alpha type-1	P18420	Psma1	0.32	0.39619
Urinary protein 1	P81827		0.99	0.02568
Desmocollin 3	A0A0G2K230	Dsc3	-0.27	0.25699
Histone cluster 1 H1 family member c	A0A0G2K654	Hist1h1c	0.98	0.03237
Complement C2	Q6MG73	C2	-0.33	0.03367
Keratin 86	A0A0G2QC11	Krt86	0.27	0.73809
Peptidyl-prolyl cis-trans isomerase A	P10111	Ppia	-0.63	0.12776
Ig-like domain-containing protein	M0R4Z4		-0.16	0.76607
Carboxypeptidase B2	Q9EQV9	Cpb2	0.26	0.13915
Proteasome subunit alpha type-5	P34064	Psma5	0.05	0.85992
Cathepsin S	Q02765	Ctss	-0.04	0.90313
Periostin	D3ZAF5	Postn	1.16	0.19404
Carboxylic ester hydrolase	Q9JKC1	Bche	0.56	0.14308
Suprabasin	F7FEM5	Sbsn	-0.72	0.10328
Group specific component	Q68FY4	Gc	0.08	0.66383
Ig-like domain-containing protein	F1M5X4		0.25	0.59172
Ectonucleotide pyrophosphatase/phosphodie	D3ZES5	Enpp2	-0.18	0.59337
Macrophage-stimulating 1	F7FMS0	Mst1	0.14	0.38180
Ig-like domain-containing protein	M0RBP7		0.29	0.49298
Fibulin-1	B1WC21	Fbln1	-0.90	0.06419
Proteasome subunit beta type-10	Q4KM35	Psmb10	-0.35	0.62600
Insulin-like growth factor-binding protein 6	P35572	Igfbp6	0.83	0.01769
Ig-like domain-containing protein	M0RBD5		0.02	0.95865
Heat shock protein HSP 90-beta	P34058	Hsp90ab1	0.44	0.43243
Proteasome subunit alpha type-6	P60901	Psma6	-0.06	0.86909
Ig-like domain-containing protein	D4A3L8		0.62	0.20691
Cathepsin Z	Q9R1T3	Ctsz	0.07	0.73059
Tubulin beta-5 chain	P69897	Tubb5	-0.89	0.01528
Complement component C1q receptor	Q9ET61	Cd93	-0.57	0.28729
Rho family-interacting cell polarization regula	Q7TP54	Ripor2	-0.16	0.78986
Heat shock protein HSP 90-alpha	P82995	Hsp90aa1	0.48	0.22243
Insulin-like growth factor-binding protein 1	P21743	Igfbp1	0.39	0.41440

Description	Accession	Gene	Log2(Fold change)	p-value
Angiogenin ribonuclease 2	Q5GAM5	Ang2	0.78	0.00683
Ribonuclease 4	O55004	Rnase4	0.67	0.02138
L-lactate dehydrogenase A chain	P04642	Ldha	-1.60	0.00794
Bridging integrator 2	Q68FR2	Bin2	-0.63	0.17241
Transthyretin	P02767	Ttr	-0.03	0.90423
Insulin-like growth factor binding protein 7, is	F1M9B2	Igfbp7	0.19	0.56586
Leucine-rich alpha-2-glycoprotein 1	Q5I0E1	Lrg1	0.18	0.58646
Copper transport protein ATOX1	Q9WUC4	Atox1	-0.23	0.25173
