# Supplementary material for: Long-term evaluation of safety and biological effects of Korean Red Ginseng (Panax Ginseng): a long-term in vivo study
Source: BMC Complement Med Ther. 2022 Nov 4;22:284. doi: 10.1186/s12906-022-03736-5 (PMC9635099; doi:10.1186/s12906-022-03736-5)
Supplement: Supplementary file 2 — Supplementary Material 2 [file 12906_2022_3736_MOESM2_ESM.pdf]

Data S2. Serum protein list in female rats administered with KRG for 12 mo.

Description	Accession	Gene	Log2(Fold change)	p-value
Complement C7	A0A0G2K7X7	C7	0.16	0.57585
Insulin-like growth factor-binding protein 3	P15473	Igfbp3	0.30	0.49321
Vascular endothelial growth factor receptor 2	O08775	Kdr	-0.15	0.64200
Apolipoprotein M	P14630	Apom	0.34	0.27474
Similar to alpha-fetoprotein	F7FAY5	LOC360919	0.06	0.79929
Apolipoprotein C-I	P19939	Apoc1	0.52	0.18766
Adenylyl cyclase-associated protein 1	Q08163	Cap1	-0.81	0.12408
Lumican	P51886	Lum	-0.11	0.76500
Immunoglobulin heavy constant mu	F1LN61	Ighm	-0.19	0.73446
Alpha-2-HS-glycoprotein	P24090	Ahsg	0.27	0.36745
Attractin	Q99J86	Atrn	0.12	0.74568
Complement C3	M0RBF1	C3	-0.09	0.76485
Ubiquitin-60S ribosomal protein L40	P62986	Uba52	-2.23	0.00529
L-lactate dehydrogenase A chain	P04642	Ldha	-1.76	0.00313
RCG49849	D3ZAE6	Vasn	0.18	0.65048
Histidine-rich glycoprotein	A0A0G2K3G0	Hrg	-0.72	0.18242
Fibrinogen-like 2	G3V7P2	Fgl2	-0.08	0.79069
Colony stimulating factor 1 receptor	A0A0G2KBC4	Csf1r	0.16	0.72038
Serotransferrin	P12346	Tf	0.90	0.01477
Glycoprotein 5, platelet	G3V9H9	NEWGENE_2724	0.98	0.01045
Serine protease inhibitor A3N	A0A0H2UHI5	Serpina3n	0.18	0.56621
Coronin-1A	Q91ZN1	Coro1a	-1.04	0.06603
Uncharacterized protein	F1LTJ5		0.17	0.62918
Ectonucleotide pyrophosphatase/phosphodiesterase family	D3ZES5	Enpp2	0.07	0.90495
Collagen, type I, alpha 2	A0A0G2KAN1	NEWGENE_621351	0.09	0.84437
Myosin heavy chain 9-like 1	G3V6P7	Myh9	-0.83	0.13173
Alpha glucosidase 2 alpha neutral subunit (Predicted)	D3ZAN3	Ganab	-0.11	0.81797
Ficolin (Collagen/fibrinogen domain containing) 1	Q5M8B4	Fcna	0.22	0.53372
Inducible T-cell co-stimulator ligand	F1LVL2	Icoslg	0.70	0.21369
Insulin-like growth factor binding protein, acid labile subur	F1LRE2	Igfals	0.30	0.42476
Adiponectin a	G3V7N9	C1qb	0.56	0.17843
Ig-like domain-containing protein	G3V8Z5		0.87	0.16601
Carboxypeptidase B2	Q9EQV9	Cpb2	0.72	0.04931
Similar to RIKEN cDNA 1300017J02	A0A0G2K896	RGD1310507	0.63	0.07592
RCG46174	G3V7P5	RGD1564614	0.21	0.47891
Ig gamma-2A chain C region	P20760	Igg-2a	0.85	0.20716
Complement factor I	A0A0G2K135	Cfi	0.31	0.31379
Serpin family F member 2	F7FHF3	Serpinf2	0.41	0.13568
14-3-3 protein zeta/delta	A0A0G2JV65	Ywhaz	-0.26	0.51977
Platelet factor 4	P06765	Pf4	0.37	0.45233
Carboxylic ester hydrolase	D3ZGK7	Ces1c	-0.45	0.25698
L-selectin	F7EY63	Sell	0.51	0.39587
Insulin-like growth factor-binding protein 5	A0A0G2JVW1	Igfbp5	0.51	0.14564
Lipopolysaccharide-binding protein	Q63313	Lbp	1.02	0.00118
Plasma kallikrein	P14272	Klkb1	0.04	0.87858
Glycosylphosphatidylinositol specific phospholipase D1, iso	G3V8B1	Gpld1	0.41	0.32724
Ig lambda-2 chain C region	P20767		0.22	0.63745
Ras-related protein Rap-1b	Q62636	Rap1b	-1.12	0.08291
Neural cell adhesion molecule 1	F1LNY3	Ncam1	-0.28	0.36428
Ig-like domain-containing protein	A0A0G2K5D2		0.21	0.73523
Keratin, type I cytoskeletal 42	Q6IFU7	Krt42	-0.64	0.29743
Insulin-like growth factor-binding protein 1	P21743	Igfbp1	-0.15	0.83647
Receptor protein-tyrosine kinase	G3V6K6	Egfr	-0.02	0.95033
Ig-like domain-containing protein	M0RDF2		-0.39	0.67807
Complement C4B (Chido blood group)	Q6MG90	C4b	0.42	0.26703
CD44 antigen	D3ZGF1	Cd44	0.14	0.80296
Extracellular superoxide dismutase [Cu-Zn]	Q08420	Sod3	0.83	0.17558
Alpha-2-macroglobulin-like 1	D3ZS19	A2ml1	0.40	0.45925
Collagen alpha-1(I) chain	P02454	Col1a1	-0.14	0.75731
Histone H3.3	P84245	H3-3b	-2.25	0.03806

Description	Accession	Gene	Log2(Fold change)	p-value
Ig-like domain-containing protein	M0R816		0.84	0.22452
Complement component factor h-like 1	Q5I0M3	Cfhr1	-0.25	0.57595
Heterogeneous nuclear ribonucleoprotein C, isoform CRA_a	A0A0G2JXW4	Hnrnpc	0.70	0.06479
Serpin A11	Q7TPA5	Serpina11	0.24	0.58306
RCG21066	D3ZJW6	rCG_21066	0.63	0.25613
Serum paraoxonase/lactonase 3	Q68FP2	Pon3	0.72	0.19671
Serine protease inhibitor A3L	P05544	Serpina3l	-0.25	0.36270
Vascular cell adhesion protein 1	P29534	Vcam1	-0.20	0.66478
Eukaryotic translation initiation factor 4A1	Q6P3V8	Eif4a1	-2.21	0.00573
Complement component C9	Q62930	C9	0.10	0.78279
Alpha-2-glycoprotein 1, zinc	Q3B8R6	Azgp1	0.00	0.99998
Peptidase inhibitor 16	D3ZGM7	Pi16	0.43	0.24669
Keratin, type II cytoskeletal 2 epidermal	A0A0G2JWX4	Krt2	-1.19	0.19712
Keratin, type I cytoskeletal 10	Q6IFW6	Krt10	-1.33	0.08958
Ribonuclease 4	O55004	Rnase4	0.59	0.18770
Gamma-glutamyl hydrolase	Q62867	Ggh	-0.03	0.95784
Selenoprotein P	P25236	Selenop	0.39	0.16173
4F2 cell-surface antigen heavy chain	Q794F9	Slc3a2	0.45	0.25118
Elongation factor 1-alpha	M0R757	LOC100360413	-1.62	0.05019
Angiopoietin-like 3	F7FHP0	Angptl3	0.41	0.12936
Ig-like domain-containing protein	M0R7Q2		0.78	0.23859
Immunoglobulin heavy constant mu	F1LPR6	Ighm	-0.99	0.17253
Proteasome subunit beta type-6	P28073	Psmb6	0.08	0.82390
Fc fragment of IgG-binding protein	D3ZJF8	Fcgbp	0.42	0.35963
Hyaluronan-binding protein 2	Q6L711	Habp2	0.06	0.82435
Vinculin	A0A0G2K8V2	Vcl	-0.90	0.04520
Murinoglobulin-2	Q6IE52	Mug2	0.08	0.85960
HGF activator	Q5EBA7	Hgfac	-0.23	0.46038
Macrophage-stimulating 1	F7FMS0	Mst1	0.29	0.35669
Keratin, type II cytoskeletal 6A	Q4FZU2	Krt6a	-1.18	0.27683
Fibronectin	F1LST1	Fn1	-0.37	0.37956
Haptoglobin	P06866	Hp	0.32	0.40895
Cytosol aminopeptidase	Q68FS4	Lap3	0.40	0.42179
CXC chemokine RTCK1	Q99ME0	Ppbp	0.27	0.62497
Bridging integrator 2	Q68FR2	Bin2	-0.61	0.19286
Corticosteroid-binding globulin	P31211	Serpina6	-0.70	0.16675
Complement factor properdin	B0BNN4	Cfp	0.06	0.88784
Coagulation factor XIII B chain	F6Q1N1	F13b	0.40	0.27401
Serine protease inhibitor A3M	F1LR92	Serpina3m	-0.25	0.70562
Complement factor B	G3V615	Cfb	-0.12	0.80414
Apolipoprotein A-V	A0A0H2UHP7	Apoa5	0.44	0.22523
X-ray repair complementing defective repair in Chinese ha	Q6AZ64	Xrcc6	-2.12	0.00746
60S ribosomal protein L15	P61314	Rpl15	-2.04	0.01281
Globin c2	A0A0G2JSV6	Hba-a2	-1.60	0.09987
Periostin	D3ZAF5	Postn	0.61	0.26578
Metalloproteinase inhibitor 3	Q4V8L0	Timp3	-0.03	0.93958
Uncharacterized protein	M0R5J4		-0.51	0.22264
Fibrinogen-like protein 1	Q5M8C6	Fgl1	0.12	0.80289
Receptor of activated protein C kinase 1	P63245	Rack1	-2.33	0.00938
Group specific component	Q68FY4	Gc	0.12	0.69805
Insulin-like growth factor-binding protein 4	P21744	Igfbp4	0.98	0.04949
Protein S (Alpha), isoform CRA_b	M0R5R0	Pros1	0.38	0.18836
Ig-like domain-containing protein	A0A0G2K9Z5		-0.37	0.62667
T-complex protein 1 subunit beta	Q5XIM9	Cct2	-2.23	0.02455
Beta-2-microglobulin	P07151	B2m	0.39	0.54078
Transgelin-2	Q5XFX0	Tagln2	-0.40	0.41927
Heparin cofactor 2	A0A0G2K8K3	Serpind1	-0.70	0.38002
40S ribosomal protein S26	A0A0G2JY64		-1.83	0.01690
CD84 molecule	A0A0G2K3N2	Cd84	0.16	0.69448
C1r protein	B5DEH7	C1r	0.31	0.49545
Insulin-like growth factor-binding protein 6	P35572	Igfbp6	0.22	0.62955

Description	Accession	Gene	Log2(Fold change)	p-value
RCG35022, isoform CRA_b	G3V6B7	Flt4	0.89	0.20502
Lymphocyte cytosolic protein 1	Q5XI38	Lcp1	-0.07	0.83999
Extracellular matrix protein 1	Q62894	Ecm1	0.47	0.29472
Dentin sialophosphoprotein	Q62598	Dspp	0.26	0.61206
Prohibitin	P67779	Phb	-2.05	0.00988
Serine protease inhibitor A3K	P05545	Serpina3k	-0.55	0.28488
Thymosin beta-4	P62329	Tmsb4x	-1.47	0.01079
Protein Z, vitamin K-dependent plasma glycoprotein	G3V8K8	Proz	-0.33	0.38391
T-kininogen 2	P08932		0.07	0.85596
RCG55135, isoform CRA_b	G3V852	Tln1	-0.58	0.22485
Alpha-1-macroglobulin	Q63041	A1m	0.25	0.33698
40S ribosomal protein SA	P38983	Rpsa	-2.06	0.01794
Histone H2B	D4A817	Hist2h2be	-1.00	0.30061
Leukemia inhibitory factor receptor	G3V7K2	Lifr	-0.13	0.73330
Ig-like domain-containing protein	F1LWW1		-1.51	0.03868
Biotinidase	A0A140TAI2	Btd	-0.04	0.89120
Ig-like domain-containing protein	M0RAV0		0.92	0.10993
ATP synthase subunit beta, mitochondrial	P10719	Atp5f1b	-1.73	0.03212
Ig-like domain-containing protein	A0A0G2K4K2		-0.11	0.87241
Keratin, type II cytoskeletal 1	Q6IMF3	Krt1	-0.87	0.29399
Mannan-binding lectin serineptidase 2	A2VCV7	Masp2	-0.76	0.15686
Coagulation factor V	A0A0G2K3W2	F5	0.29	0.40305
Apolipoprotein H	Q5I0M1	Apoh	-0.09	0.75608
Cathepsin L1	P07154	Ctsl	0.87	0.06666
Growth hormone receptor	A0A0G2K423	Ghr	0.41	0.40456
Serum albumin	P02770	Alb	0.35	0.51372
Complement C1r subcomponent-like protein	F1LP96	C1rl	0.01	0.95758
Complement C8 gamma chain	D3ZPI8	C8g	-0.25	0.31893
Ig-like domain-containing protein	M0R7M5		-0.40	0.70677
Threonine--tRNA ligase 1, cytoplasmic	Q5XHY5	Tars1	-2.66	0.01221
Keratin, type I cytoskeletal 17	A0A0G2K9Q9	Krt17	-0.96	0.25216
Lysozyme C-1	P00697	Lyz1	0.73	0.24173
Uncharacterized protein	D3ZM33		-1.84	0.02570
Complement factor H-related protein B	A0A0G2JYC4	LOC100361907	-0.06	0.86530
Keratin, type II cytoskeletal 8	Q10758	Krt8	-0.56	0.57006
Keratin, type I cytoskeletal 25	Q6IFX0	Krt25	0.62	0.17880
Carboxypeptidase N subunit 2	F1LQT4	Cpn2	0.17	0.75508
Ig-like domain-containing protein	F1M0U4		-0.36	0.52049
60S ribosomal protein L18a	P62718	Rpl18a	-2.06	0.02614
60 kDa heat shock protein, mitochondrial	P63039	Hspd1	-1.15	0.12319
Adhesion G protein-coupled receptor F5	Q9WVT0	Adgrf5	0.07	0.85477
Plasminogen	Q01177	Plg	-0.20	0.50866
Keratin, type II cytoskeletal 5	Q6P6Q2	Krt5	-1.56	0.18861
Insulin-like growth factor I	A0A0G2JX40	Igf1	0.29	0.55131
Ac1873	Q7TQ70	Fga	0.72	0.23799
Platelet-activating factor acetylhydrolase	Q5M7T7	Pla2g7	-0.02	0.97292
Anionic trypsin-1	P00762	Prss1	0.24	0.41417
Complement component C6	Q811M5	C6	1.86	0.05748
Secreted phosphoprotein 24	A0A0G2K9X1	Spp2	-0.66	0.32407
Inter-alpha trypsin inhibitor, heavy chain 1	B2RYM3	Itih1	0.47	0.18987
Keratin, type II cytoskeletal 75	Q6IG05	Krt75	-0.45	0.70289
All-trans-retinol dehydrogenase [NAD(+)] ADH7	G3V7J9	Adh7	-0.33	0.45074
Peroxiredoxin-2	A0A0G2JSH9	Prdx2	-0.72	0.20894
Serum paraoxonase/arylesterase 1	P55159	Pon1	-0.86	0.22345
Prothrombin	G3V843	F2	0.28	0.44989
Programmed cell death protein 6	G3V7W1	Pdcd6	-1.58	0.08411
Clusterin	G3V836	Clu	-0.09	0.80232
Protein Z-dependent protease inhibitor	Q62975	Serpina10	0.26	0.50988
Lecithin cholesterol acyltransferase	O35849	Lcat	0.83	0.06810
Coagulation factor XI	A0A0G2K4I9	F11	-0.46	0.37204
Trypsin	P00761		0.11	0.83602

Description	Accession	Gene	Log2(Fold change)	p-value
Thrombospondin 1	M0R979	Thbs1	0.30	0.35842
Apolipoprotein N	Q5M890	Apon	0.33	0.27548
Ferritin heavy chain	P19132	Fth1	-0.81	0.11676
Coagulation factor XIII A chain	G3V811	F13a1	1.04	0.09942
Heat shock 70 kDa protein 1A	P0DMW0	Hspa1a	-1.53	0.05984
Angiotensinogen	P01015	Agt	0.19	0.64181
Immunoglobulin joining chain	G3V6G1	Jchain	0.33	0.46623
Leukocyte cell-derived chemotaxin 2	D4A526	Lect2	0.65	0.05064
Glutathioneroxidase 1	P04041	Gpx1	-0.24	0.57924
Beta-enolase	P15429	Eno3	-0.78	0.10528
Hemopexin	P20059	Hpx	-0.03	0.91888
Coactosin-like protein	B0BNA5	Cotl1	-1.27	0.06877
Alpha-amylase	E9PSQ1	Amy1a	-0.57	0.41350
Ig-like domain-containing protein	D3ZQR5		0.25	0.80299
alpha-1,2-Mannosidase	A0A0G2JW29	Man1a1	0.09	0.82855
Proteasome subunit alpha type-6	P60901	Psma6	-0.35	0.22327
Ig-like domain-containing protein	D3ZAB3		-0.24	0.77260
Coagulation factor XII	D3ZTE0	F12	0.00	0.99893
Polymeric immunoglobulin receptor	A0A0G2K5U5	Pigr	-0.45	0.40418
Carboxypeptidase	Q6AYS3	Ctsa	0.25	0.59717
Cytochrome c, somatic	P62898	Cycs	0.17	0.76868
Adiponectin, C1Q and collagen domain-containing	A0A0G2K845	Adipoq	-0.85	0.32882
Leucine-rich alpha-2-glycoprotein 1	Q5I0E1	Lrg1	1.04	0.07436
T-kininogen 1	P01048	Map1	0.19	0.60896
Immunoglobulin heavy constant mu	F1LM30	Ighm	-0.11	0.81240
Ig-like domain-containing protein	F1LYQ4		0.81	0.27854
Proteasome subunit alpha type-1	P18420	Psma1	0.00	0.99816
Tubulin alpha-1B chain	Q6P9V9	Tuba1b	-1.06	0.07407
Peroxiredoxin-5, mitochondrial	Q9R063	Prdx5	-1.45	0.04958
Pulmonary surfactant-associated protein D	P35248	Sftpd	0.59	0.25388
Ig-like domain-containing protein	A0A0G2K6T8		0.49	0.29052
Complement C2	Q6MG73	C2	0.37	0.27490
CD163 antigen (Predicted)	D3Z9U2	Cd163	0.04	0.92173
Insulin-like growth factor II	P01346	Igf2	0.20	0.70056
Tubulin alpha-4A chain	Q5XIF6	Tuba4a	-1.04	0.08683
Heat shock cognate 71 kDa protein	P63018	Hspa8	-0.56	0.23460
Ig-like domain-containing protein	M0R9U2		0.97	0.14729
Out at first protein homolog	Q6AYE5	Oaf	0.34	0.31583
Multimerin 1	D4A3E0	Mmrn1	-0.31	0.56106
Keratin 16	Q6IFU9	Krt16	-0.80	0.41653
Complement component C8 beta chain	P55314	C8b	-1.56	0.03726
Ig-like domain-containing protein	A0A0G2K0N6		1.04	0.32390
Histone H1.4	P15865	Hist1h1e	-0.86	0.39394
Ig-like domain-containing protein	F1M5X4		0.40	0.57338
Cadherin-2	G3V803	Cdh2	0.28	0.42780
Alpha-1B-glycoprotein	Q9EPH1	A1bg	0.65	0.21038
Ig-like domain-containing protein	D3ZMS7		0.55	0.58304
C-reactive protein	P48199	Crp	0.16	0.72404
Spectrin beta chain	G3V6S0	Sptbn1	0.24	0.46420
Histone H4	P62804	H4c2	-1.44	0.22790
Ig-like domain-containing protein	M0RDL2		2.00	0.03695
Protein/nucleic acid deglycase DJ-1	O88767	Park7	0.20	0.55047
Ig-like domain-containing protein	A0A0G2JW41		1.41	0.05825
Ig-like domain-containing protein	D4ACR1		-0.14	0.77646
Phospholipase A2, membrane associated	P14423	Pla2g2a	-0.29	0.60863
Peptidoglycan recognition protein 2	M0R485	Pglyrp2	0.19	0.56388
Alpha-1-inhibitor 3	P14046	A1i3	0.21	0.44826
Similar to Vanin-3 (Predicted)	D4A183	Vnn3	0.32	0.33257
Podocalyxin	A0A0G2K2L1	Podxl	-0.16	0.73927
Carboxypeptidase N catalytic chain	Q9EQV8	Cpn1	-0.09	0.85045
Fetuin-B	Q9QX79	Fetub	-0.21	0.69178

Description	Accession	Gene	Log2(Fold change)	p-value
Ig-like domain-containing protein	M0RBK4		1.16	0.03159
Complement C8 alpha chain	D3ZWD6	C8a	0.27	0.36490
Complement factor H	G3V9R2	Cfh	-0.20	0.69217
Hemoglobin subunit beta-2	P11517		-0.41	0.58428
Plexin domain containing 2 (Predicted)	B5DEZ8	Plxdc2	-0.18	0.75091
Fibulin-1	B1WC21	Fbln1	-1.08	0.13859
Uncharacterized protein	G3V9J1		0.04	0.91579
Proteasome subunit alpha type-4	P21670	Psma4	0.11	0.85468
Alpha-tropomyosin 3	Q63607	Tpm1	-1.28	0.05029
Ig-like domain-containing protein	A0A0G2JX36		0.02	0.98245
Ig gamma-2B chain C region	P20761	Igh-1a	0.60	0.22605
Phosphoglycerate kinase 1	P16617	Pgk1	-0.54	0.23447
Junction plakoglobin	Q6P0K8	Jup	0.37	0.70705
von Willebrand factor	F1M957	Vwf	-0.42	0.21965
Ig-like domain-containing protein	A0A0G2K5X3		-0.29	0.72171
Heterogeneous nuclear ribonucleoproteins A2/B1	A7VJC2	Hnrnpa2b1	-1.26	0.12216
Ig-like domain-containing protein	M0R8G6		1.85	0.00984
DEAH (Asp-Glu-Ala-His) box polypeptide 9 (Predicted)	D4A9D6	Dhx9	-1.66	0.03960
Tubulin beta chain	M0R8B6	Tubb1	-0.66	0.18625
Inter-alpha-trypsin inhibitor heavy chain H3	D3ZBS2	Itih3	-0.34	0.63132
Pleckstrin	A0A0G2K393	Plek	0.00	0.99714
Profilin-1	P62963	Pfn1	-0.81	0.15604
Proteasome subunit beta type-7	Q9JHW0	Psmb7	-0.32	0.36444
Ig-like domain-containing protein	F1M663		0.98	0.13443
Hepcidin	Q99MH3	Hamp	0.35	0.39861
Insulin receptor-related protein	Q64716	Insrr	0.23	0.68900
Thyroxine-binding globulin	A0A140TAB0	Serpina7	0.12	0.80083
Carboxylic ester hydrolase	G3V7J5	Ces2e	-0.27	0.66536
Heterogeneous nuclear ribonucleoprotein H	G3V9Q3	Hnrnph1	-0.19	0.70304
Tubulin beta chain	G3V7C6	Tubb4b	-0.61	0.20294
Proteoglycan 4	F1LRA5	Prg4	-0.14	0.62502
Glutathioneroxidase	A0A0G2K531	Gpx3	-0.38	0.47821
Complement component C1q receptor	Q9ET61	Cd93	-0.18	0.73596
Ig-like domain-containing protein	D3ZE00		0.50	0.53221
Urinary protein 2	P81828		0.60	0.46393
Retinol-binding protein 4	P04916	Rbp4	-0.36	0.29824
Cystatin E/M	Q8VHC1	Cst6	0.09	0.85750
Alpha-1-acid glycoprotein	P02764	Orm1	-0.46	0.49758
Ig-like domain-containing protein	A0A0G2JV42		1.20	0.11020
Glia-derived nexin	P07092	Serpine2	0.46	0.42223
Moesin	A0A096MK30	Msn	0.05	0.88262
Ceruloplasmin	G3V7K3	Cp	-0.11	0.75001
Myeloperoxidase	A0A0G2K1A2	Mpo	-0.11	0.76975
Ig-like domain-containing protein	F1M4R1		-0.12	0.83751
Retinoic acid receptor responder (Tazarotene induced) 2	Q5BK77	Rarres2	-0.46	0.33811
RCG62531, isoform CRA_g	A0A140TAF0	Tpm3	-0.07	0.92026
Nucleoside diphosphate kinase B	P19804	Nme2	-0.70	0.18621
Interleukin-1 receptor accessory protein	F1M9B9	Il1rap	0.01	0.97268
Ig-like domain-containing protein	F1M229		-0.25	0.74258
Apolipoprotein C-III	A0A0G2K8Q1	Apoc3	0.61	0.20799
Ig-like domain-containing protein	F1LW26		1.55	0.02845
Ig-like domain-containing protein	M0R628		0.36	0.64844
Ig-like domain-containing protein	M0RBP7		0.16	0.77814
Tropomyosin alpha-4 chain	P09495	Tpm4	-1.97	0.00743
C4b-binding protein beta chain	A0A5C5	C4bpb	-0.12	0.81240
Carbonic anhydrase 2	P27139	Ca2	0.09	0.89442
Maltase-glucoamylase	D3ZTX4	Mgam	-0.14	0.69065
Ig-like domain-containing protein	D3ZPL2		0.70	0.09570
Proteasome subunit alpha type-5	P34064	Psma5	-0.91	0.02530
Plasma protease C1 inhibitor	Q6P734	Serping1	-0.02	0.95259
Cathepsin S	Q02765	Ctss	0.12	0.71975

Description	Accession	Gene	Log2(Fold change)	p-value
Desmocollin 1	A0A0G2KA90	Dsc1	-0.39	0.62952
Xaa-Pro aminopeptidase 2	Q99MA2	Xpnpep2	0.30	0.31674
Complement factor D	P32038	Cfd	-0.11	0.86919
Alpha-mannosidase	Q6P762	Man2b1	0.33	0.23582
Afamin	G3V9R9	Afm	-0.31	0.50283
Glyceraldehyde-3-phosphate dehydrogenase	P04797	Gapdh	-0.60	0.07154
Vimentin	P31000	Vim	-1.35	0.05750
Similar to BC049975 protein	F1M8F5	LOC100909605	-0.56	0.54005
Ig-like domain-containing protein	A0A0G2K9W1		-0.42	0.65932
Desmoplakin	F1LMV6	Dsp	-0.97	0.30458
Filamin A	C0JPT7	Flna	-1.27	0.01760
Phospholipid transfer protein	E9PSP1	Pltp	0.62	0.22071
Actin, cytoplasmic 1	A0A0G2K3K2	Actb	-1.48	0.06106
Keratin, type II cytoskeletal 80	Q6IMF1	Krt80	-0.43	0.53606
Peptidyl-prolyl cis-trans isomerase B	P24368	Ppib	-0.34	0.44573
CD59 glycoprotein	P27274	Cd59	0.15	0.74020
Similar to Ig variable region, light chain	F1M7I8	RGD1565617	0.78	0.23991
Transforming growth factor, beta-induced	D4A8G5	Tgfbi	-0.86	0.15323
Apolipoprotein A-IV	P02651	Apoa4	0.22	0.48317
Proprotein convertase subtilisin/kexin type 9	P59996	Pcsk9	0.31	0.57545
ATP synthase subunit alpha	F1LP05	Atp5f1a	-0.25	0.71488
Complement C4	P08649	C4	-0.13	0.70444
Ig-like domain-containing protein	D3ZEP5		1.52	0.03573
Apolipoprotein C-II (Predicted)	G3V8D4	Apoc2	0.60	0.23834
Complement C1s subcomponent	Q6P6T1	C1s	0.46	0.64216
14-3-3 protein beta/alpha	P35213	Ywhab	-0.73	0.10947
Ig-like domain-containing protein	M0RA79		1.08	0.15792
Calponin (Fragment)	D3ZRX9	Cnn2	-0.68	0.12027
Complement C1q subcomponent subunit C	P31722	C1qc	-0.35	0.56449
Plasminogen activator inhibitor 2 type A	P29524	Serpinb2	-0.39	0.14391
Protein AMBP	Q64240	Ambp	0.05	0.90069
Ig gamma-2C chain C region	P20762		0.54	0.31269
Apolipoprotein A-I	P04639	Apoa1	0.55	0.11135
Ig-like domain-containing protein	A0A0G2JXP0		0.58	0.25720
Annexin A2	Q07936	Anxa2	-1.12	0.08266
Ig-like domain-containing protein	M0RE02		1.30	0.11712
Methanethiol oxidase	F1LRJ9	Selenbp1	0.30	0.38038
Afamin	P36953	Afm	-1.14	0.13926
Aspartate aminotransferase, cytoplasmic	P13221	Got1	0.21	0.46165
Ig-like domain-containing protein	D3ZZ08		0.53	0.41419
Keratin, type II cytoskeletal 72	A0A0G2K7N1	Krt72	-0.62	0.56728
Cysteine and glycine-rich protein 1	P47875	Csrp1	-0.19	0.69709
Ig-like domain-containing protein	A0A0G2K458		1.28	0.06549
Alpha-2 antiplasmin	Q80ZA3	Serpinf1	0.06	0.85750
Serine (Or cysteine)ptidase inhibitor, clade C (Antithrombin	Q5M7T5	Serpinc1	0.00	0.99187
Zyxin	D4A7U1	Zyx	-0.44	0.33567
Fructose-bisphosphate aldolase A	P05065	Aldoa	-0.05	0.89794
Di-N-acetylchitobiase	Q01460	Ctbs	-0.09	0.81502
Apolipoprotein A-II	P04638	Apoa2	0.46	0.35950
Ig-like domain-containing protein	A0A0G2JZN1		0.35	0.61667
Pyruvate kinase PKM	P11980	Pkm	0.68	0.13967
Ig-like domain-containing protein	A0A0G2K7S9		1.19	0.04373
Serine (Or cysteine) proteinase inhibitor, clade A (Alpha-1 a	Q5M8C3	Serpina4	-0.03	0.95137
Uncharacterized protein	F1LTN6		-0.04	0.95141
Inter-alpha-trypsin inhibitor heavy chain 2	D3ZFH5	Itih2	-0.20	0.55953
Chromogranin-A	P10354	Chga	0.15	0.67973
Metalloproteinase inhibitor 2	P30121	Timp2	-0.90	0.14546
Uncharacterized protein	F1LWS4	Cfhr2	0.29	0.29775
Protein C, isoform CRA_b	F7FMY6	Proc	0.37	0.33624
Thrombospondin 1	A0A0G2JV24	Thbs1	-0.46	0.34991
Murinoglobulin-1	Q03626	Mug1	0.02	0.94109

Description	Accession	Gene	Log2(Fold change)	p-value
Ig delta chain C region (Fragment)	P01883		0.20	0.76672
Multiple inositol polyphosphate phosphatase 1	G3V7H2	Minpp1	-0.36	0.39671
Hemoglobin subunit beta-1	P02091	Hbb	-1.10	0.27375
Cofilin-1	P45592	Cfl1	-0.05	0.92211
Urinary protein 1	P81827		0.56	0.46259
Triggering receptor-expressed on myeloid cells-like 1	D3ZYT6	Treml1	-0.56	0.25461
RCG21069	D3ZCD6	rCG_21069	0.43	0.39175
Ig-like domain-containing protein	F1M2W3		1.71	0.04612
Lipocln_cytosolic_FA-bd_dom domain-containing protein	F1M6Y6		-0.20	0.62698
Superoxide dismutase [Cu-Zn]	P07632	Sod1	0.12	0.83298
D-3-phosphoglycerate dehydrogenase	O08651	Phgdh	-1.00	0.05860
Carboxylic ester hydrolase	Q9JKC1	Bche	0.21	0.47417
Ig kappa chain C region, A allele	P01836		1.62	0.02383
Cathepsin B	Q6IN22	Ctsb	-0.31	0.38247
Tsukushin	Q6QMY6	Tsku	0.40	0.32669
Proteasome subunit alpha type	A0A0G2K0W9	Psma7	-0.58	0.28384
CD5 antigen-like	Q4KM75	Cd5l	-0.11	0.80685
Ig-like domain-containing protein	M0R8B5		1.64	0.05916
Ig-like domain-containing protein	D3ZHM9		1.30	0.05718
Apolipoprotein C-IV	P55797	Apoc4	0.83	0.20665
Complement C1q subcomponent subunit A	P31720	C1qa	-0.45	0.25086
Tropomyosin alpha-3 chain	Q63610	Tpm3	-0.60	0.34903
Serum amyloid A protein	Q5M878	Saa4	0.65	0.25296
RCG61833	F1LQS6	Xdh	-0.12	0.76065
Regulator of G-protein signaling 18	Q4L0E8	Rgs18	-0.70	0.37390
Ig-like domain-containing protein	A0A0G2JXB7		-0.11	0.79269
LIM and SH3 domain protein 1	Q99MZ8	Lasp1	0.31	0.59555
Ig-like domain-containing protein	A0A0G2JXF0		0.85	0.07202
Ig-like domain-containing protein	F1LTY5		1.79	0.02152
Extracellular link domain-containing 1 (Predicted)	D3ZD19	Lyve1	0.38	0.53072
Globin a1	Q62669	LOC103694855	-0.17	0.79081
Apolipoprotein B-100	F1M6Z1	Apob	0.53	0.16069
Rho GDP-dissociation inhibitor 1	Q5XI73	Arhgdia	-0.42	0.41333
Insulin-like growth factor 2 receptor	G3V824	Igf2r	-0.27	0.58020
Transthyretin	P02767	Ttr	-0.55	0.23919
Tyrosine-protein kinase Mer	P57097	Mertk	-0.91	0.25448
Ig-like domain-containing protein	F1M5L5		1.01	0.11355
Ig-like domain-containing protein	F1LWD0		-0.06	0.86166
Keratin 83	A7M746	Krt83	1.15	0.04508
Proteasome subunit beta type-1	P18421	Psmb1	-1.11	0.00207
Pro-neuropeptide Y	P07808	Npy	1.22	0.09982
Proteasome subunit beta	G3V7Q6	Psmb5	0.12	0.82543
Ig-like domain-containing protein	F1LZY6		1.40	0.14430
Ig-like domain-containing protein	A0A0G2K7I1		0.29	0.63047
FYN-binding protein 1	D3ZIE4	Fyb1	0.22	0.65189
Carboxylesterase 1C	P10959	Ces1c	-0.04	0.93945
Hemoglobin subunit alpha-1/2	P01946	Hba1	0.72	0.18905
Alpha-1-antiproteinase	A0A0G2JZ73	Serpina1	0.15	0.75745
Coagulation factor IX	P16296	F9	-0.67	0.17823
Suprabasin	F7FEM5	Sbsn	-0.70	0.35805
Apolipoprotein E	P02650	Apoe	1.13	0.09644
Peptidyl-prolyl cis-trans isomerase A	P10111	Ppia	-1.06	0.15222
Ig-like domain-containing protein	M0RBD5		0.34	0.51086
Heat shock protein HSP 90-beta	P34058	Hsp90ab1	0.17	0.71529
Inter alpha-trypsin inhibitor, heavy chain 4	Q5EBC0	Itih4	-0.09	0.82884
Ig-like domain-containing protein	F1LXY6		0.55	0.22629
Carboxypeptidase Q	Q6IRK9	Cpq	-0.35	0.46775
Alpha-2-macroglobulin	P06238	A2m	-0.07	0.84023
Serum amyloid P-component	P23680	Apcs	0.06	0.87432
Ig-like domain-containing protein	D3ZFF8		0.04	0.94839
C4b-binding protein alpha chain	Q63514	C4bpa	-0.95	0.06824

Description	Accession	Gene	Log2(Fold change)	p-value
Tubulin-specific chaperone A	Q6PEC1	Tbca	-0.82	0.11153
Protein S100-A4	P05942	S100a4	-0.02	0.94836
Ig-like domain-containing protein	D4A3D1		0.09	0.90495
Transferrin receptor protein 1	G3V679	Tfrc	0.15	0.83099
Zinc finger and BTB domain containing 7a	G3V8P6	Zbtb7a	0.61	0.37527
Ig-like domain-containing protein	D3ZWC1		1.59	0.02912
Mannose-binding protein C	P08661	Mbl2	0.69	0.17639
Antigen p97 (Melanoma associated) identified by monoclo	D4ADK7	Meltf	1.35	0.33735
Ig-like domain-containing protein	A0A0G2K1F0		0.60	0.22700
Ig-like domain-containing protein	F1M1R0	LOC690813	0.31	0.42681
Rho family-interacting cell polarization regulator 2	Q7TP54	Ripor2	-0.65	0.22889
PDZ and LIM domain protein 1	P52944	Pdlim1	-0.45	0.50021
ADF-H domain-containing protein	D4A315		0.13	0.83332
Plexin B2	D3ZQ57	Plxnb2	-0.15	0.79080
Kininogen-1	P08934	Kng1	-0.55	0.06670
C-type lectin domain family 3, member B	D3ZUU6	Clec3b	-0.28	0.38177
Protein S100-A9	A0A0H2UHJ1	S100a9	0.05	0.92082
Ig-like domain-containing protein	D4ADK9		0.69	0.37149
Keratin 86	A0A0G2QC11	Krt86	1.30	0.01176
Cystatin-C	P14841	Cst3	0.12	0.83708
Thioredoxin domain-containing protein	A0A0G2K3Z9		-0.39	0.26936
Integrin beta-3	Q8R2H2	Itgb3	-0.42	0.30297
Endoplasmic reticulum chaperone BiP	P06761	Hspa5	-0.99	0.03154
Glycoprotein Ib platelet subunit alpha	D3ZQU7	Gp1ba	-0.08	0.89072
Ig-like domain-containing protein	M0RD98		2.87	0.00395
Ig-like domain-containing protein	M0RBX3		0.04	0.96017
Proteasome subunit beta type-10	Q4KM35	Psmb10	-0.38	0.61787
Vitronectin	Q3KR94	Vtn	-0.42	0.27076
RCG28243	D3ZQV0	Prss3	-0.99	0.18573
Fatty acid-binding protein, liver	P02692	Fabp1	0.61	0.37082
Coagulation factor X	Q63207	F10	-1.15	0.02275
Catalase	P04762	Cat	-0.80	0.11359
Complement C5	A0A096P6L9	C5	-0.36	0.61214
Ig-like domain-containing protein	F1MAE7		-0.32	0.63592
Heat shock protein HSP 90-alpha	P82995	Hsp90aa1	0.12	0.86389
Mannose-binding protein A	P19999	Mbl1	-0.71	0.25396
Ig-like domain-containing protein	A0A0G2JZV7		0.27	0.61990
Ig-like domain-containing protein	F1LZ11		1.08	0.12000
Gelsolin	Q68FP1	Gsn	-0.91	0.13411
Prosaposin	F7EPE0	Psap	-0.36	0.30022
