# Supplementary material for: Long-term evaluation of safety and biological effects of Korean Red Ginseng (Panax Ginseng): a long-term in vivo study
Source: BMC Complement Med Ther. 2022 Nov 4;22:284. doi: 10.1186/s12906-022-03736-5 (PMC9635099; doi:10.1186/s12906-022-03736-5)
Supplement: Supplementary file 3 — Supplementary Material 3 [file 12906_2022_3736_MOESM3_ESM.docx]

**Table S1.** Relative organ weight of rats in the 4, 8, and 12-month oral administration of Korean Red Ginseng (KRG) in male and female rats, expressed as mean ± SD (*p* < 0.05).

| **Male** | **Relative weight (%)** | | | | | | | | | | | | | |  |
| --- | --- | --- | --- | --- | --- | --- | --- | --- | --- | --- | --- | --- | --- | --- | --- |
| **Month** | **Dose (mg/kg)** | **Liver** | **Spleen** | **Kidney (R)** | **Kidney (L)** | **Adrenal gland (R)** | **Adrenal gland (L)** | **Thymus** | **Brain** | **Lung** | **Testis (R)** | **Testis (L)** | **Epididymis (R)** | **Epididymis (L)** | |
| 4 | Control | 2.472 ± 0.125 | 0.167 ± 0.017 | 0.320 ± 0.021 | 0.330 ± 0.021 | 0.005 ± 0.001 | 0.005 ± 0.001 | 0.054 ± 0.015 | 0.432 ± 0.031 | 0.473 ± 0.068 | 0.457 ± 0.038 | 0.458 ± 0.044 | 0.178 ± 0.015 | 0.177 ± 0.013 | |
|  | 300 | 2.547 ± 0.103 | 0.168 ± 0.020 | 0.350 ± 0.029 | 0.389 ± 0.144 | 0.006 ± 0.001 | 0.006 ± 0.001 | 0.059 ± 0.010 | 0.455 ± 0.043 | 0.490 ± 0.037 | 0.465 ± 0.050 | 0.473 ± 0.050 | 0.190 ± 0.023 | 0.187 ± 0.021 | |
| 8 | Control | 2.410 ± 0.297 | 0.151 ± 0.029 | 0.299 ± 0.018 | 0.294 ± 0.019 | 0.005 ± 0.000 | 0.006 ± 0.002 | 0.060 ± 0.009 | 0.389 ± 0.030 | 0.441 ± 0.046 | 0.420 ± 0.056 | 0.422 ± 0.053 | 0.152 ± 0.012 | 0.160 ± 0.016 | |
|  | 300 | 2.974 ± 0.579 | 0.176 ± 0.041 | 0.354 ± 0.062 | 0.361 ± 0.071 | 0.005 ± 0.001 | 0.005 ± 0.001 | 0.053 ± 0.014 | 0.374 ± 0.017 | 0.427 ± 0.040 | 0.427 ± 0.019 | 0.416 ± 0.015 | 0.162 ± 0.013 | 0.190 ± 0.075 | |
| 12 | Control | 3.036 ± 0.333 | 0.199 ± 0.026 | 0.348 ± 0.044 | 0.362 ± 0.043 | 0.005 ± 0.001 | 0.006 ± 0.001 | 0.032 ± 0.015 | 0.350 ± 0.019 | 0.363 ± 0.027 | 0.364 ± 0.027 | 0.361 ± 0.030 | 0.112 ± 0.018 | 0.115 ± 0.009 | |
|  | 300 | 2.783 ± 0.457 | 0.177 ± 0.038 | 0.329 ± 0.066 | 0.328 ± 0.057 | 0.007 ± 0.001 | 0.006 ± 0.001 | 0.044 ± 0.014 | 0.350 ± 0.012 | 0.353 ± 0.027 | 0.344 ± 0.089 | 0.395 ± 0.038 | 0.11 ± 0.035 | 0.126 ± 0.013 | |
| **Female** | **Relative weight (%)** | | | | | | | | | | | | |  | |
| **Month** | **Dose (mg/kg)** | **Liver** | **Spleen** | **Kidney (R)** | **Kidney (L)** | **Adrenal gland (R)** | **Adrenal gland (L)** | **Thymus** | **Brain** | **Lung** | **Uterus** | **Ovary (R)** | **Ovary (L)** |  | |
| 4 | Control | 2.545 ± 0.158 | 0.205 ± 0.012 | 0.356 ± 0.037 | 0.344 ± 0.034 | 0.013 ± 0.001 | 0.014 ± 0.001 | 0.080 ± 0.016 | 0.690 ± 0.054 | 0.658 ± 0.081 | 0.260 ± 0.085 | 0.026 ± 0.005 | 0.023 ± 0.005 |  | |
|  | 300 | 2.610 ± 0.160 | 0.205 ± 0.015 | 0.361 ± 0.021 | 0.354 ± 0.036 | 0.012 ± 0.001 | 0.013 ± 0.002 | 0.082 ± 0.018 | 0.673 ± 0.031 | 0.667 ± 0.123 | 0.254 ± 0.094 | 0.018 ± 0.008* | 0.018 ± 0.005* |  | |
| 8 | Control | 2.511 ± 0.164 | 0.186 ± 0.014 | 0.371 ± 0.096 | 0.332 ± 0.033 | 0.012 ± 0.002 | 0.013 ± 0.002 | 0.047 ± 0.007 | 0.637 ± 0.035 | 0.691 ± 0.130 | 0.322 ± 0.112 | 0.027 ± 0.004 | 0.034 ± 0.008 |  | |
|  | 300 | 2.386 ± 0.099 | 0.202 ± 0.028 | 0.355 ± 0.037 | 0.340 ± 0.027 | 0.011 ± 0.003 | 0.012 ± 0.002 | 0.050 ± 0.012 | 0.640 ± 0.022 | 0.687 ± 0.095 | 0.328 ± 0.098 | 0.029 ± 0.006 | 0.029 ± 0.007 |  | |
| 12 | Control | 2.329 ± 0.244 | 0.179 ± 0.025 | 0.330 ± 0.032 | 0.320 ± 0.030 | 0.011 ± 0.001 | 0.010 ± 0.007 | 0.036 ± 0.009 | 0.585 ± 0.064 | 0.456 ± 0.048 | 0.370 ± 0.149 | 0.015 ± 0.003 | 0.016 ± 0.003 |  | |
|  | 300 | 2.276 ± 0.173 | 0.198 ± 0.069 | 0.306 ± 0.021 | 0.340 ± 0.087 | 0.011 ± 0.002 | 0.009 ± 0.001 | 0.035 ± 0.007 | 0.552 ± 0.052 | 0.467 ± 0.043 | 2.034 ± 3.147 | 0.015 ± 0.002 | 0.014 ± 0.003 |  | |
