# Supplementary material for: Long-term evaluation of safety and biological effects of Korean Red Ginseng (Panax Ginseng): a long-term in vivo study
Source: BMC Complement Med Ther. 2022 Nov 4;22:284. doi: 10.1186/s12906-022-03736-5 (PMC9635099; doi:10.1186/s12906-022-03736-5)
Supplement: Supplementary file 4 — Supplementary Material 4 [file 12906_2022_3736_MOESM4_ESM.docx]

**Table S2.** Summary of histological findings after Korean Red Ginseng (KRG) treatment using minimal, slight, moderate, and severe grades.

| **Organ** | **Sex** |  | **Male** | | **Female** | |
| --- | --- | --- | --- | --- | --- | --- |
|  | **Group** |  | **G1** | **G2** | **G1** | **G2** |
|  | **No. of animals** |  | **6** | **6** | **6** | **6** |
|  | **Findings** | **Grade** |  |  |  |  |
| **Epididymis** | Aspermia, luminal, unilateral | Slight | 0 | 2 | - | - |
|  |  | Total Number of affected | **0** | **2** | - | - |
|  |  |  |  |  |  |  |
| **Kidney** | Chronic progressive nephropathy | Minimal | 0 | 2 | 6 | 4 |
|  |  | Slight | 4 | 2 | 0 | 0 |
|  |  | Moderate | 2 | 2 | 0 | 0 |
|  |  | Total Number of affected | **6** | **6** | **6** | **4** |
|  |  |  |  |  |  |  |
| **Liver** | Bile duct hyperplasia, focal | Slight | 0 | 0 | 0 | 2 |
|  | Infiltration, mononuclear cell, focal | Minimal | 0 | 0 | 2 | 2 |
|  |  | Total Number of affected | **0** | **0** | **2** | **4** |
|  |  |  |  |  |  |  |
| **Lung** | Alveolar macrophage aggregation | Slight | 0 | 0 | 2 | 0 |
|  |  | Total Number of affected | **0** | **0** | **2** | **0** |
|  |  |  |  |  |  |  |
| **Ovary** | Atrophy, age-related | Slight | - | - | 6 | 6 |
|  |  | Total Number of affected | **-** | **-** | **6** | **6** |
|  |  |  |  |  |  |  |
| **Testis** | Atrophy, tubular, nonspecific, unilateral | Slight | 0 | 2 | - | - |
|  |  | Total Number of affected | **0** | **2** | **-** | **-** |
|  |  |  |  |  |  |  |
| **Uterus** | Dilatation, lumen | Present | - | - | 0 | 2 |
|  | Squamous metaplasia, uterine gland | Minimal | - | - | 6 | 4 |
|  |  | Total Number of affected | **-** | **-** | **6** | **6** |
| When three animals were investigated instead of six, the values obtained from the three animals were multiplied by two. | | | | | | |
