# Supplementary material for: Long-term evaluation of safety and biological effects of Korean Red Ginseng (Panax Ginseng): a long-term in vivo study
Source: BMC Complement Med Ther. 2022 Nov 4;22:284. doi: 10.1186/s12906-022-03736-5 (PMC9635099; doi:10.1186/s12906-022-03736-5)
Supplement: Supplementary file 5 — Supplementary Material 5 [file 12906_2022_3736_MOESM5_ESM.docx]

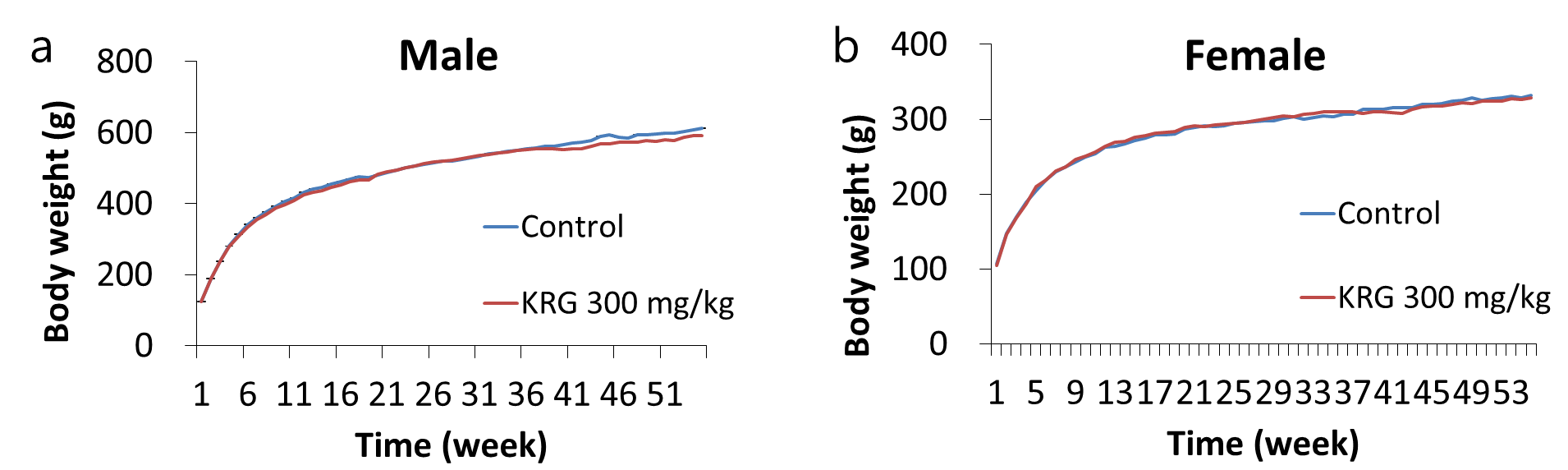


**Fig. S1.** Body weight changes in male (a) and female (b) rats in the 12-mo oral administration test. A total of 300 mg/kg of Korean Red Ginseng (KRG) was administered to Sprague Dawley rats for up to 12 mo.
