# Supplementary material for: Long-term evaluation of safety and biological effects of Korean Red Ginseng (Panax Ginseng): a long-term in vivo study
Source: BMC Complement Med Ther. 2022 Nov 4;22:284. doi: 10.1186/s12906-022-03736-5 (PMC9635099; doi:10.1186/s12906-022-03736-5)
Supplement: Supplementary file 6 — Supplementary Material 6 [file 12906_2022_3736_MOESM6_ESM.docx]

**
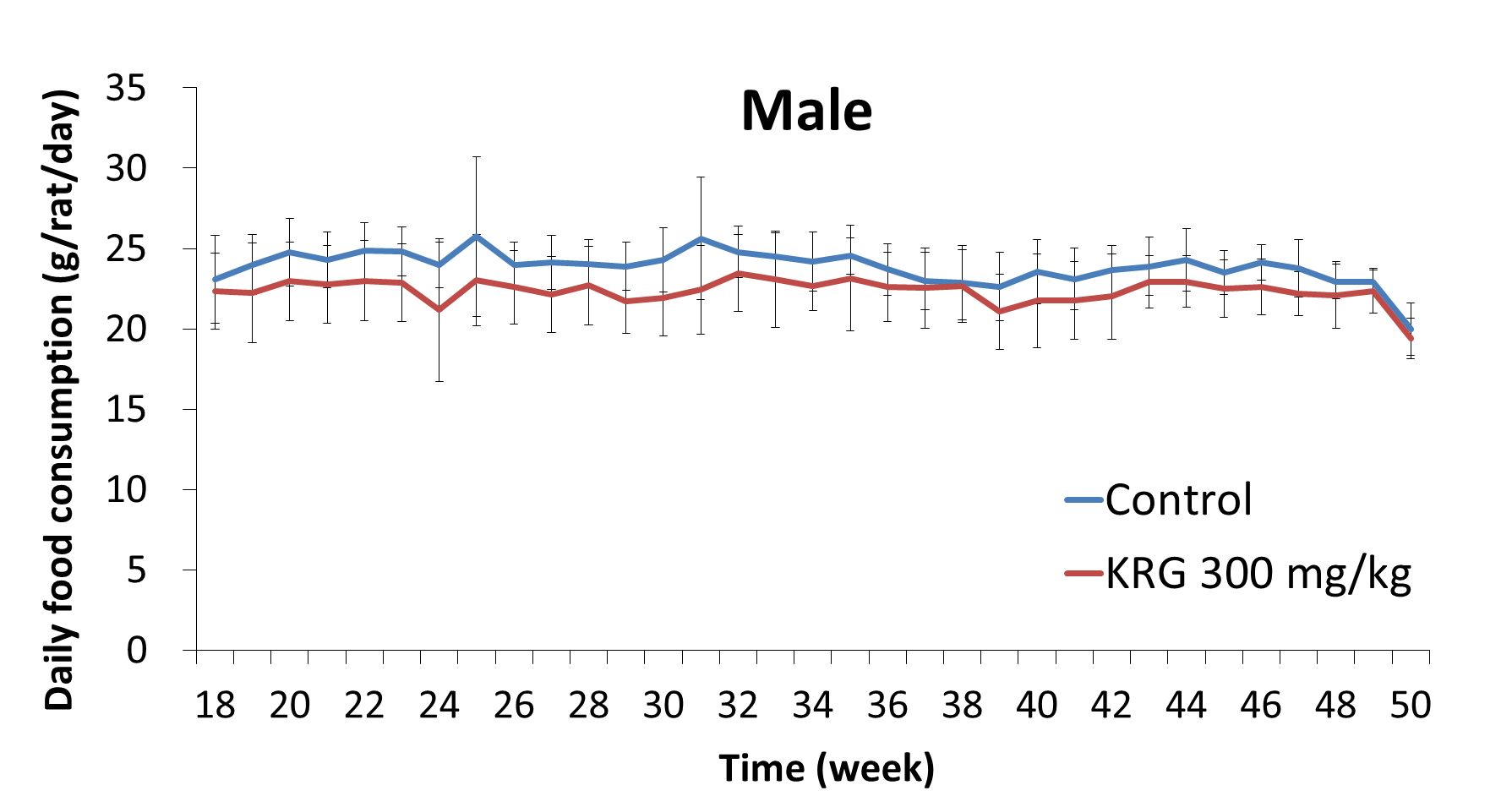
**a

**
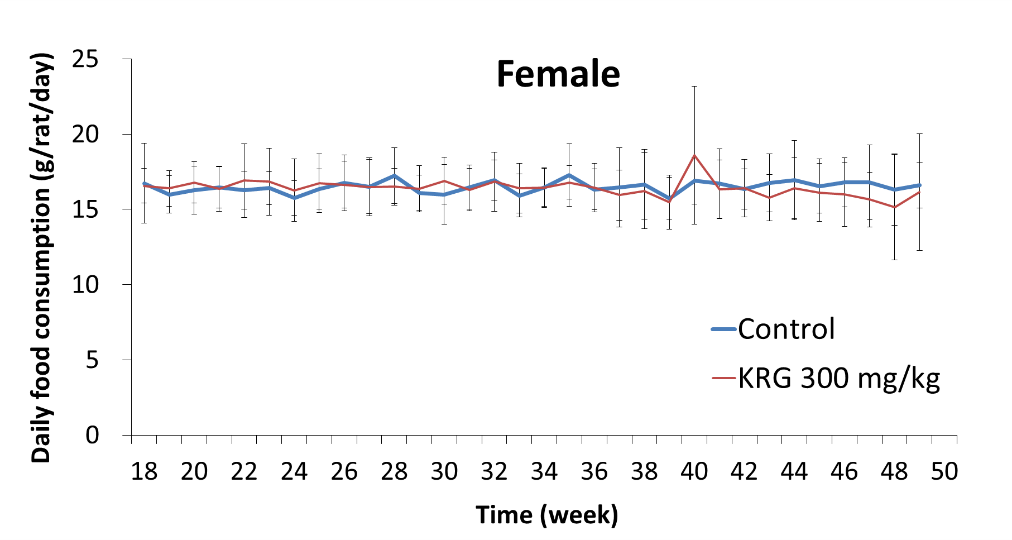
**

b

**Fig. S2.** Food consumption changes in male (a) and female (b) rats in the 12-mo oral administration test. Food consumption was measured weekly during the mid and late experimental study from weeks 18 to 50.
